# Experimental colonization with *Blastocystis* ST4 is associated with protective immune responses and modulation of gut microbiome in a DSS-induced colitis mouse model

**DOI:** 10.1007/s00018-022-04271-9

**Published:** 2022-04-18

**Authors:** Lei Deng, Lukasz Wojciech, Chin Wen Png, Eileen Yiling Koh, Thet Tun Aung, Dorinda Yan Qin Kioh, Eric Chun Yong Chan, Benoit Malleret, Yongliang Zhang, Guangneng Peng, Nicholas Robert John Gascoigne, Kevin Shyong Wei Tan

**Affiliations:** 1 Laboratory of Molecular and Cellular Parasitology, Healthy Longevity Translational Research Programme and Department of Microbiology and Immunology, Yong Loo Lin School of Medicine, National University of Singapore, 5 Science Drive 2, Singapore, 117545 Singapore; 2 The Key Laboratory of Animal Disease and Human Health of Sichuan Province, College of Veterinary Medicine, Sichuan Agricultural University, Chengdu, 611130 Sichuan China; 3 Department of Microbiology and Immunology, Immunology Translational Research Programme, Yong Loo Lin School of Medicine, Immunology Programme, Life Sciences Institute, National University of Singapore, Singapore, 117597 Singapore; 4 Department of Pharmacy, Faculty of Science, National University of Singapore, 5 Science Drive 2, Singapore, 117545 Singapore; 5 Singapore Immunology Network (SIgN), A*STAR, 8A Biomedical Grove, Immunos Building, Singapore, 138648 Singapore

**Keywords:** *Blastocystis*, Gut microbiota, Th2, Treg, Colitis

## Abstract

**Background:**

*Blastocystis* is a common gut protistan parasite in humans and animals worldwide, but its interrelationship with the host gut microbiota and mucosal immune responses remains poorly understood. Different murine models of *Blastocystis* colonization were used to examine the effect of a common *Blastocystis* subtype (ST4) on host gut microbial community and adaptive immune system.

**Results:**

*Blastocystis* ST4-colonized normal healthy mice and *Rag1*^−/−^ mice asymptomatically and was able to alter the microbial community composition, mainly leading to increases in the proportion of *Clostridia* vadinBB60 group and *Lachnospiraceae* NK4A136 group, respectively. *Blastocystis* ST4 colonization promoted T helper 2 (Th2) response defined by interleukin (IL)-5 and IL-13 cytokine production, and T regulatory (Treg) induction from colonic lamina propria in normal healthy mice. Additionally, we observed that *Blastocystis* ST4 colonization can maintain the stability of bacterial community composition and induce Th2 and Treg immune responses to promote faster recovery from experimentally induced colitis. Furthermore, fecal microbiota transplantation of *Blastocystis* ST4-altered gut microbiome to colitis mice reduced the severity of colitis, which was associated with increased production of short-chain fat acids (SCFAs) and anti-inflammatory cytokine IL-10.

**Conclusions:**

The data confirm our hypothesis that *Blastocystis* ST4 is a beneficial commensal, and the beneficial effects of *Blastocystis* ST4 colonization is mediated through modulating of the host gut bacterial composition, SCFAs production, and Th2 and Treg responses in different murine colonization models.

**Supplementary Information:**

The online version contains supplementary material available at 10.1007/s00018-022-04271-9.

## Background

*Blastocystis*, classified under the stramenopile phylum, is the most common unicellular intestinal parasite found in humans and various animals, with an estimate of more than 1 billion people colonized worldwide [1]. The clinical significance of *Blastocystis* remains controversial, although it has been widely studied for more than 100 years [2]. *Blastocystis* has been associated with inflammatory bowel disease (IBD) and irritable bowel syndrome (IBS) [3, 4]. Some microbiome studies have reported that *Blastocystis* colonization decreases the abundance of beneficial bacterial *Bifidobacterium* in humans and mouse models [5, 6]. However, most *Blastocystis*–gut microbiota association studies have revealed that *Blastocystis*-colonized individuals have a higher gut bacterial diversity and lower levels of *Bacteroides* compared to *Blastocystis*-free individuals, suggesting that *Blastocystis* may be a beneficial commensal rather than a pathogen [7, 8]. These discrepancies may be influenced by the complex nature of *Blastocystis* wherein several genetically distinct subtypes exist [1]. Different subtypes exhibit extensive differences in genome size, effects on gut microbiota, and immune responses [8–10]. However, there is still no consensus on the existence of pathogenic and non-pathogenic subtypes to date, although some in vitro* Blastocystis*–host studies reveal subtype-associated pathobiological outcomes [5, 11].

Based on analyses of the small subunit (SSU) rRNA gene, 25 subtypes have been identified in humans and a wide range of animals [12]. Among them, ST1–4 are the most reported subtypes in humans, accounting for around 90% of human infected cases [13]. The prevalence of ST4 appears to be more geographically variable, as it is mainly reported in Europe and rarely found in South America, Africa and Asia [14, 15]. Interactions among host, gut microbiota, and *Blastocystis* can drive the development of the immune system in colonized individuals and play a role in maintaining intestinal homeostasis. Indeed, it has been reported in vitro and in mouse models that *Blastocystis* is involved in the host’s innate and adaptive immunity in regulating the function and differentiation of the immune cell repertoire of the gut [16, 17]. T helper 2 (Th2) cells and T regulatory (Treg) cells are well known for their crucial roles in fighting extracellular parasite infection and suppressing intestinal inflammation [18]. However, it is unclear whether these immune cells are involved in the process of *Blastocystis* colonization.

Although several studies have investigated the association between *Blastocystis* and the gut microbial composition, only limited studies have analyzed this association at the subtype level. *Blastocystis* ST4 was originally isolated from a Wistar rat [19] and is the most prevalent subtype observed in the Flemish Gut Flora Project (FGFP) [8], TwinsUK [20] and American Gut Project (AGP) [21]. In the current study, we explored the interactions between *Blastocystis* ST4, gut microbiota, and host immunity in different mice models. Furthermore, we evaluated the impact of *Blastocystis* ST4 colonization on experimentally induced colitis. Our findings showed that *Blastocystis* ST4 colonization not only alters the gut microbial composition, but also enhances the accumulation of Th2 and Treg cells in the colonic mucosa. Additionally, *Blastocystis* ST4 colonization prevents loss of microbiota diversity and contributes towards attenuation of disease in a murine model of experimental colitis. These results revealed a previously unrecognized mutualistic relationship between *Blastocystis* ST4, gut microbiota and host immunity.

## Methods

### Culture of *Blastocystis*

The axenized *Blastocystis* isolate WR1 belonging to ST4 was used in this study. ST4-WR1 was originally isolated from a healthy Wistar rat during an animal survey at National University of Singapore [19]. ST4 is a common zoonotic subtype frequently detected in humans and a wide range of animals, including nonhuman primates, bovines, goats, dogs, rodents, and birds [22]. *Blastocystis* was maintained in 10 ml of pre-reduced Iscove’s modified Dulbecco’s medium (IMDM) (Gibco) supplemented with heat-inactivated 10% horse serum (Gibco). Cultures were incubated under anaerobic conditions in an Anaerojar (Oxoid) with gas pack (Oxoid) at 37 °C and subcultured every 3–4 days. *Blastocystis* cell counts were assessed manually using hemocytometer (Kova International).

### Mice and treatments

The animal experiments were performed according to the Singapore National Advisory Committee for Laboratory Animal Research guidelines. All animal procedures performed in this study were approved by the Institutional Animal Care and Use Committee of National University of Singapore. C57BL/6 and *Rag1*^*−/−*^ (*Rag1*^*tm1Mom*^) mice of 8–12 weeks of age were bred and maintained in the animal facilities of the National University of Singapore (NUS). Littermates of the same sex and age were randomly assigned to the different experimental groups. Mice were colonized with *Blastocystis* ST4 via oral gavage with 5 × 10^7^ live *Blastocystis* cells suspended in sterile phosphate-buffered saline (PBS) three times per week before euthanization at day 3 post last gavage. The control mice were orally gavaged with equal amounts of PBS at the same time. The mice used in all the experiments were age and sex matched.

For dextran sulfate sodium (DSS)-induced colitis with ST4 colonization model: C57BL/6 mice were administered 2% DSS (molecular mass = 36,000–50,000 Da; MP Biomedicals) w/v in drinking water for 7 days. Mice were then orally gavaged with 5 × 10^7^ live *Blastocystis* cells three times per week for two consecutive weeks. In all experiments, mice were monitored daily for changes in body weight, stool consistency and presence of fecal blood. Disease activity index (DAI) was used to assess the severity of colitis as previously described [23]. In brief, daily calculation of DAI for each mouse was based on weight loss, occult blood, and stool consistency/diarrhea. Each parameter was scored on a scale of 1–4, with a maximum DAI score of 12. Score 0: no weight loss, normal stool, no blood; score 1: 1–3% weight loss, softer stool; score 2: 3–6% weight loss, loose stool, blood visible in stool; score 3: 6–9% weight loss, diarrhea, blood visible in stool; score 4: > 9% weight loss, diarrhea, gross bleeding.

### Scanning electron microscopy

Mouse cecum and colon tissues were processed by opening the gut longitudinally without disturbing the intestinal contents with the help of binocular dissecting microscope. The opened tissues were pinned down to a silicone mat in four corners and fixed in 2.5% glutaraldehyde at 4 °C overnight as previously described [24]. The overnight fixed samples were washed two times (20 min each) with PBS and kept at 4 °C until further processing. Afterward, they were processed by post-fixing in 1% osmium tetroxide for 1 h, followed by dehydration with increasing concentrations of ethanol and critical-point dried. The dried samples were coated with 25 nm of gold and imaged on a field emission JSM-6701F Scanning Electron Microscopy (SEM) at a voltage of 10 kV.

### DNA extraction and real-time quantitative PCR

DNA of the mouse fecal microbiota was isolated by using QIAamp Fast DNA Stool Mini Kit (Qiagen, Germany) according to the manufacturers. Real-time quantitative PCR (qPCR) to estimate the number of *Blastocystis* cells in feces was performed in accordance with a previously published protocol [25]. Briefly, 2 µl of the extracted DNA was added to a mixture of 5 µl SsoAdvanced Universal SYBR Green Supermix and 0.5 μM forward primer BL18SPPF1 (5‵- AGTAGTCATACGCTCGTCTCAAA -3‵) and 0.5 μM reverse primer BL18SR2PP (5‵- TCTTCGTTACCCGTTACTGC -3‵) for the SSU rRNA gene of *Blastocystis.* The qPCR was performed on an ABI 7500 real-time PCR system instrument (Life Technologies) at 95 °C for 5 min followed by 45 cycles of 95 °C for 15 s, 68 °C for 10 s, and 72 °C for 15 s, and completed with melting curve analysis. Each sample was quantified in triplicate. A standard curve was produced with a dilution series (10^7^, 10^6^, 10^5^, 10^4^, 10^3^, 10^2^, 10^1^ cells/ml) of *Blastocystis* ST4. The Ct values of each sample were compared with that of the standard curve and the number of *Blastocystis* cells was calculated.

All mice were examined for the presence of *Blastocystis* by SEM and qPCR amplification of the *Blastocystis* SSU rRNA gene. Mice were considered to be successfully colonized after *Blastocystis* was observed in the gut lumen by SEM, and SSU rRNA gene was successfully amplified from the stool samples.

### Fecal microbiota transplantation

Fecal microbiota transplantation (FMT) was performed through oral gavage of feces preparations from donor mice as previously described [26]. In brief, microbiota for FMT was obtained from control (PBS-gavaged) *Rag1*^*−/−*^ mice or ST4-colonized *Rag1*^*−/−*^ mice administered with *Blastocystis* ST4. Feces were collected, diluted with PBS (50 mg/ml), and then administered to recipients by oral gavage (10 mg/mice) three times a week.

### Histology

For histological studies, the small intestine (SI), cecum and colon tissues were fixed in 4% neutral buffered formalin before processing and embedding in paraffin based on standard protocols. 4.5 μm sections were prepared and stained with hematoxylin and eosin (H&E). Histology scoring was performed in a blinded fashion, whereby changes in intestinal crypt architecture, level of tissue damage, goblet cell loss, and inflammatory cell infiltrates were scored as previously described [27].

### 16S rRNA gene sequencing

The V3–V4 region of the 16S rRNA gene was amplified using the 341-F (CCTAYGGGRBGCASCAG) and 806-R (GGACTACNNGGGTATCTAAT) primers. Gene amplification was carried out using Phusion High-Fidelity PCR Master Mix (New England Biolabs). Single amplifications were performed in 50 μl reactions with 50 ng of template DNA. Cycling protocol consisted of 94 °C for 4 min, followed by 30 cycles of 94 °C for 30 s, 54 °C for 30 s, and 72 °C for 30 s, with a final extension of 72 °C for 5 min. The size of the amplicons was determined using 2% agarose gel electrophoresis. Samples with size between 400 and 450 bp were extracted and purified from agarose gel using Qiagen Gel Extraction kit (Qiagen, Germany). Sequencing libraries were generated from the amplicons using NEBNext Ultra DNA Library Pre^®^ Kit for Illumina, following manufacturer's recommendations and index codes were added. The library quality was assessed on the Qubit@ 2.0 Fluorometer (Thermo Scientific) and Agilent Bioanalyzer 2100 system. Qualified library was sequenced on an Illumina Novaseq platform (Illumina, San Diego CA, USA) and 250 bp paired-end reads were generated.

### *Bioinformatic* and statistical analysis

Paired-end reads were assigned to samples based on their unique barcodes and truncated by cutting off the barcode and primer sequences. Paired-end clean reads were merged using FLASH version 1.2.7, which was designed to merge paired-end reads when the reads overlap the read generated from the opposite end of the same DNA fragment [28], and the splicing sequences were called tags. The tags were compared with the reference database using UCHIME algorithm to detect chimera sequences [29], and these chimera sequences were subsequently removed, resulting in effective tags. Next, effective tags were trimmed to 200 bp to remove the low-quality portion of the sequences (mean quality score < 20) using the DADA2 plugin for Quantitative Insights into Microbial Ecology (QIIME2 version 2021.02) [30, 31]. Taxonomic assignment was performed using the BLAST fitted classifier trained on the SILVA 138 reference database with the feature-classifier plugin for QIIME2 [32] based on 100% similarity. Biodiversity index analysis was calculated using QIIME2 and displayed with R software (Version R-4.0.3). Alpha diversity analysis was done using the metrics Shannon, Simpson, and Richness index. Pairwise comparisons of microbial communities in different groups were carried out using permutational multivariate analysis of variance (PERMANOVA, Bray–Curtis distance) in the q2-diversity-plugin in QIIME. Principal coordinate analysis (PCoA) and heatmap analysis were performed using R package.

### Isolation of lamina propria cells

To analyze intestinal lymphocytes, the intestines were longitudinally opened and washed with ice-cold PBS to remove luminal contents. The tissues were cut into 1 cm pieces and incubated in Roswell Park Memorial Institute (RPMI) 1640 medium (Sigma-Aldrich) containing 1 mM EDTA (Sigma-Aldrich) and 1 mM DTT (Sigma-Aldrich) at room temperature for 20 min under slow rotation and spun down to remove the supernatant. The remaining pieces were incubated in RPMI containing 25% HEPES, 10% fetal calf serum (FCS), 1 mM EDTA, and 1 mM DTT at 37 ℃ for 1 h under slow rotation and then washed by PBS to remove epithelial cells and intraepithelial lymphocytes. Tissue pieces were digested with 0.3 mg/ml collagenase D (Sigma-Aldrich), 0.4 mg/ml dispase (Gibco) and 40 µg/ml DNase I (Roche) at 37 ℃ for 30 min under slow rotation. The digested tissue pieces and supernatants were filtered by 70 μm cell strainer and glass wool separately. After centrifugation, pellets containing the lamina propria (LP) lymphocytes were harvested.

### Flow cytometric analysis

Lymphocytes were stimulated for 6 h with a cell stimulation cocktail of PMA (50 ng/ml), ionomycin (750 ng/ml) and 0.7 μl/ml GolgiStop (monensin, BD Biosciences). Live/dead stain was used to evaluate the viability of the cells. For surface staining, stimulated cells were stained with anti-CD4 (APC/FITC; Biolegend). Fixation and permeabilization buffers from Biolegend were used for intracellular cytokine staining. Fixed and permeabilized cells were stained with fluorochrome-conjugated anti-mouse antibodies against interleukin (IL)-4 (BV421; Biolegend), IL-5 (PE; Biolegend), IL-13 (PE; Biolegend), IL-10 (BV421; Biolegend), IL-17 (PerCP-Cy5.5; eBioscience, CA, USA), interferon gamma (IFN-γ) (BV711; Biolegend), and tumor necrosis factor (TNF-α) (APC; eBioscience) at 4 ℃ for 10 h. Flow cytometric analysis was performed on Fortessa X-20 (BD biosciences) and the data were analyzed using FlowJo_V10 software. The gating strategies are shown in Supplementary Fig. 4.

### LC/MS/MS assay

Liquid chromatography/tandem mass spectrometry (LC/MS/MS) was carried out for analysis of short-chain fatty acids (SCFAs) in derivatized stool extracts as previously described [33]. In brief, 500 μl of ice-cold extraction solvent containing 10 μM of d_5_-benzoic acid as internal standard (IS) was added to 250 mg of stool sample and subjected to vortex mixing for 5 min at ambient temperature. The suspension was then centrifuged at 18,000*g* for 10 min at 4 °C. The supernatant was carefully removed and centrifuged again at 18,000*g* for 10 min at 4 °C. An aliquot of 100 μl was subsequently derivatized using a final concentration of 10 mM aniline and 5 mM EDC for 2 h at 4 °C. The derivatization reaction was quenched using a final concentration of 18 mM succinic acid and 4.6 mM 2-mercaptoethanol for 2 h at 4 °C. All samples were stored at 4 °C until analysis on the same day. Analysis was performed using an Agilent 1290 Infinity LC system (Agilent Technologies, Santa Clara, CA, USA) interfaced with an AB Sciex QTRAP 5500 hybrid linear ion-trap quadrupole mass spectrometer equipped with a TurboIonSpray source (Applied Biosystems, Foster City, CA). Details of the LC/MS/MS and calibration methods were similar as previously described [33].

### Statistical analysis

Statistical analysis was performed using R-4.0.3 software and GraphPad Prism 8 software (GraphPad Software, CA, USA). Two independent replicates were performed for each experiment. The unpaired two-tailed Student’s *t* test was used to evaluate differences between two groups. Two-way ANOVA and one-way ANOVA analysis with Tukey multiple comparisons test was used for comparison of more than two groups. Graphs show mean ± SEM. * *p* < 0.05, ** *p* < 0.01, *** *p* < 0.001.

## Results

### *Blastocystis* ST4 colonization exerts no harmful effects on normal healthy mice

**Fig. 1:**
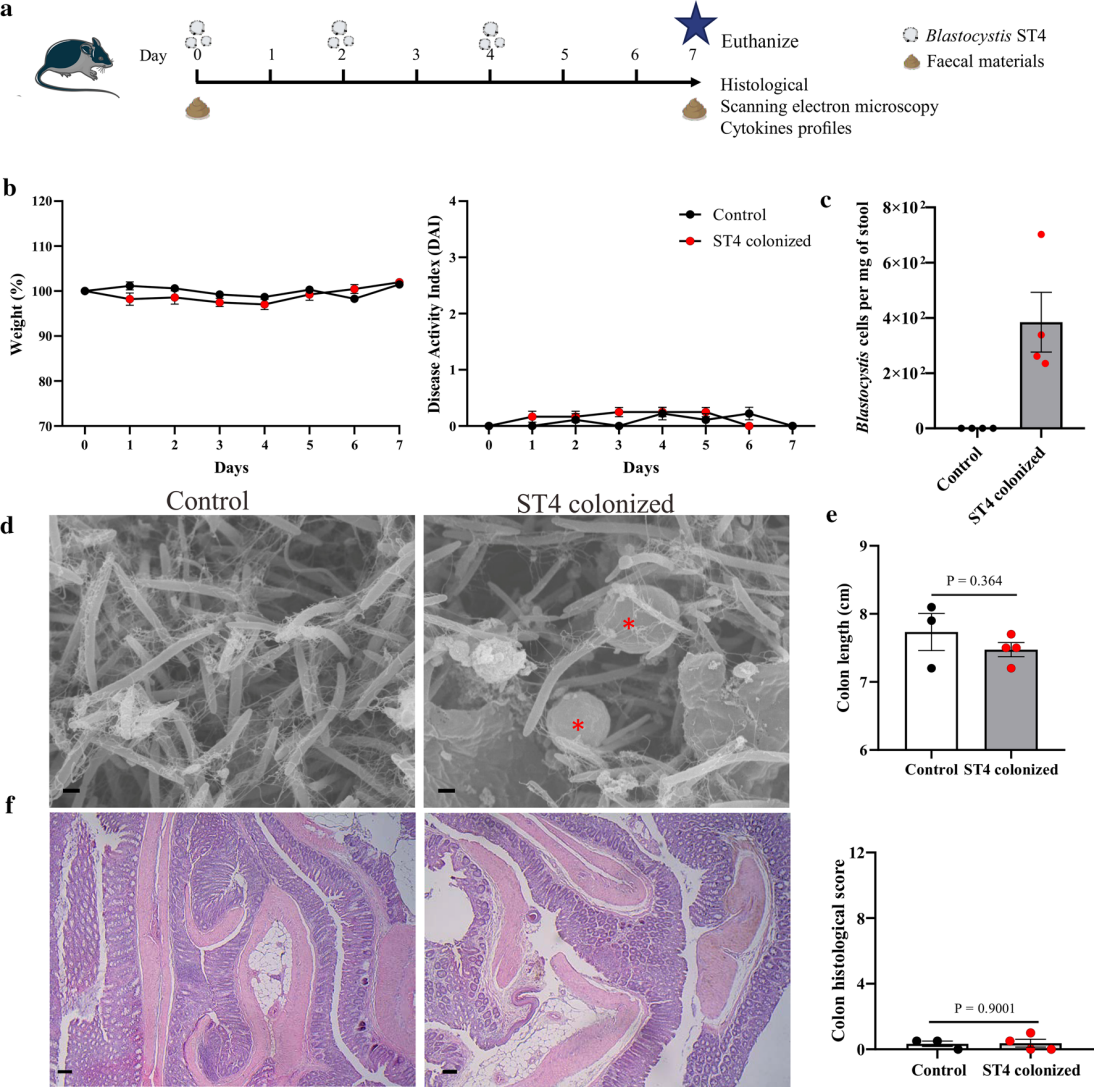
*Blastocystis* ST4 colonization did not induce any abnormal effects on normal healthy mice. **a** Experimental design. **b** Weight changes and disease activity index (DAI) between control and ST4-colonized mice. **c** *Blastocystis* ST4 cells per milligram of stool in ST4-clonized mice. **d** Scanning electron microscopy (SEM) of colonic and cecum tissues from control and ST4-clonized mice, *Blastocystis* are indicated with red asterisk (*). Scale bar = 1 μm. **e** Colon length at endpoint. **f** Representative micrographs of H&E-stained colon sections from control and ST4-colonized mice, and colonic histological scores at day 7. Scale bar = 100 μm

*Blastocystis* ST4 is commonly found in humans and a variety of animals worldwide, predominantly in rats [34]. Although some in vitro studies have shown that ST4 infection can increase epithelial permeability and release pro-inflammatory cytokines [16, 35], the effects of ST4 in vivo are less well understood. To determine the effects of *Blastocystis* ST4 colonization on host intestinal bacterial communities and immune responses, we established a mouse model of oral *Blastocystis* colonization. Specifically, C57BL/6 mice were orally inoculated with 5 ×10^7^ cells of *Blastocystis* three times per week, and the mice were euthanized 3 days after the final gavage with *Blastocystis* (Fig. 1a). Interestingly, we observed that *Blastocystis* ST4 colonization did not cause any abnormalities, characterized by no significant difference in weight change and DAI between ST4-colonized and non-colonized control mice (Fig. 1b). The number of *Blastocystis* ST4 cells was quantitated by qPCR analysis (Fig. 1c), and scanning electron microscopic analysis of the contents of the cecum and colon from ST4-clonized mice revealed the presence of *Blastocystis*, which colonizes the intestinal lumen and closely adheres to intestinal microbes (Fig. 1d). Additionally, we examined the histopathology of SI, cecum and colon, and scored these based on the degree of inflammation and tissue damage. The mice colonized with ST4 showed intact mucosal epithelium without any ulcerated lesions or an abnormal level of inflammatory cell infiltration (Fig. 1e, f; Supplementary Fig. 1), which is consistent with previous findings in rats experimentally colonized with *Blastocystis* ST4 [17, 36]. Overall, these findings indicate that *Blastocystis* ST4 colonization did not cause abnormal phenotypic changes or any gut histopathology within the duration of the experiment.

### *Blastocystis* ST4 colonization alters the bacterial community composition in normal healthy mice

**Fig. 2:**
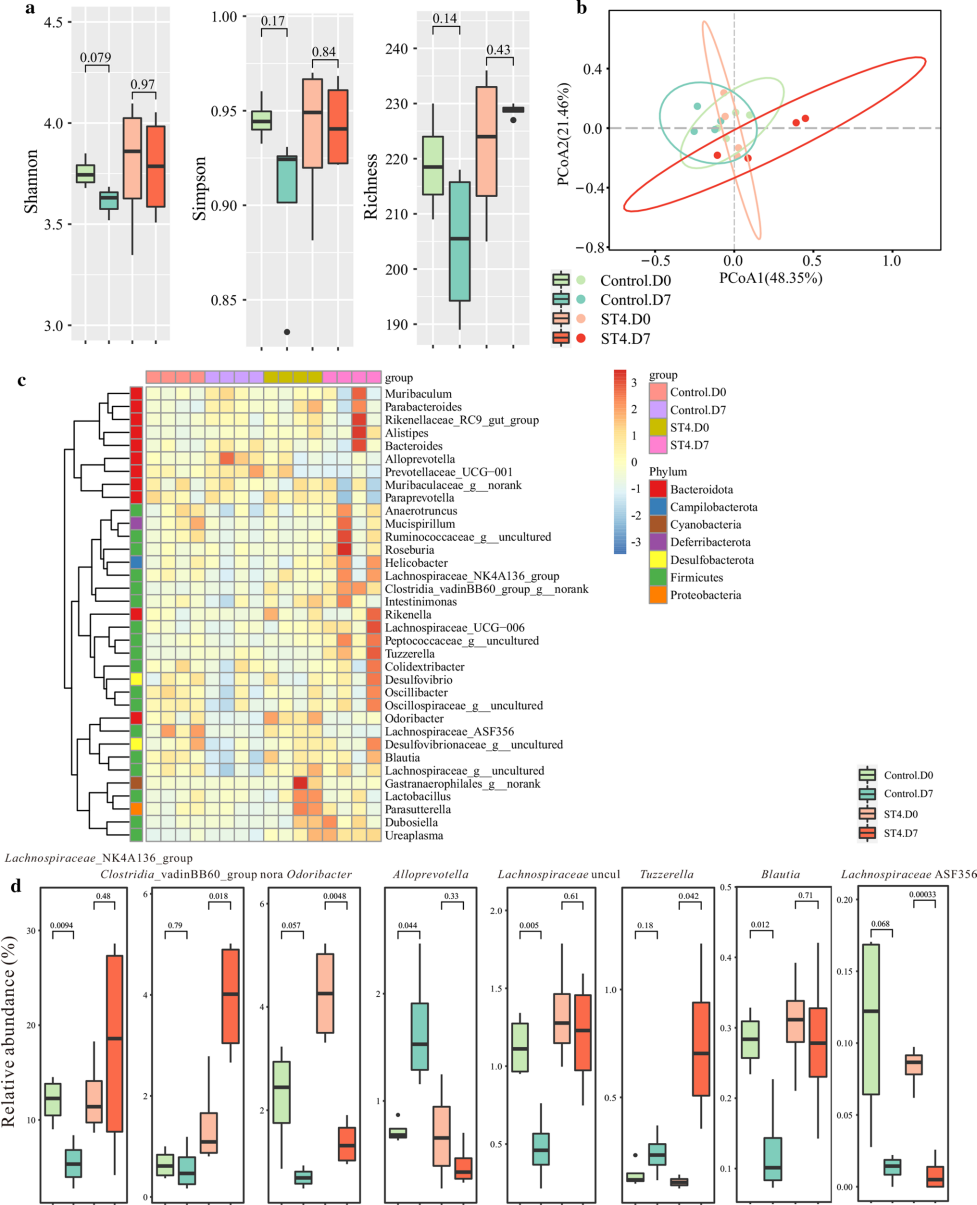
*Blastocystis* ST4 colonization alters the murine fecal bacterial community compositions. **a** Alpha diversity was measured by Shannon, Simpson, and Richness indexes in the fecal samples of control and ST4-colonized mice (*n* = 4 mice per group). **b** PCoA of fecal gut microbiota in control and ST4-clonized mice at day 0 and day 7. **c** Heatmap of ST4 colonization-associated taxonomic markers at day 7. **d** Bacterial genera (relative abundance in the top 35) showing significant differences in their relative abundance between control and ST4-colonized mice

*Blastocystis*-colonized individuals showed distinct difference in bacterial community structure when compared to non-colonized individuals [37], suggesting *Blastocystis* has the ability to modulate intestinal microbiota. To investigate the effects of *Blastocystis* ST4 colonization on gut bacterial communities, fecal samples of control and ST4-colonized mice were collected at day 0 and day 7, and bacterial compositions were investigated using 16S rRNA gene sequencing. Rarefaction analysis was used to estimate the total number of observed features that could be identified from the samples; this showed that a credible number of reads (average 101, 487) had been measured in each group (Supplementary Fig. 2). No significant differences in bacterial diversity and richness were detected in ST4-colonized and control mice over time, as measured by Shannon, Simpson and Richness indices (Fig. 2a). However, we observed significant difference in bacterial community composition in the ST4-colonized group between day 0 and day 7, measured by beta diversity of Bray–Curtis dissimilarity (PERMANOVA *p* = 0.026; Fig. 2b; Supplementary Table 1). The heatmap showed that differences in the relative abundances of various taxa between control and ST4-colonized mice (Fig. 2c). Specifically, we observed higher levels of unclassified *Clostridia* vadinBB60 group, *Tuzzerella*, and *Peptococcaceae* uncultured, and lower levels of *Odoribacter*, and *Lachnospiraceae* ASF356 at day 7 post-*Blastocystis* ST4 colonization (Fig. 2d, Supplementary Fig. 3). In contrast, the significantly reduced bacterial taxa in ST4-free mice were *Lachnospiraceae* NK4A136 group, *Odoribacter*, *Lachnospiraceae* uncultured, *Blautia* and *Oscillibacter*, while *Alloprevotella*, *Bacteroides*, and *Paraprevotella* were the most significantly increased (Fig. 2d, Supplementary Fig. 3). Overall, these data indicated that *Blastocystis* ST4 colonization alters the bacterial community compositions in normal healthy mice.

### *Blastocystis* ST4 colonization induces the accumulation of Th2 and Treg cells

**Fig. 3:**
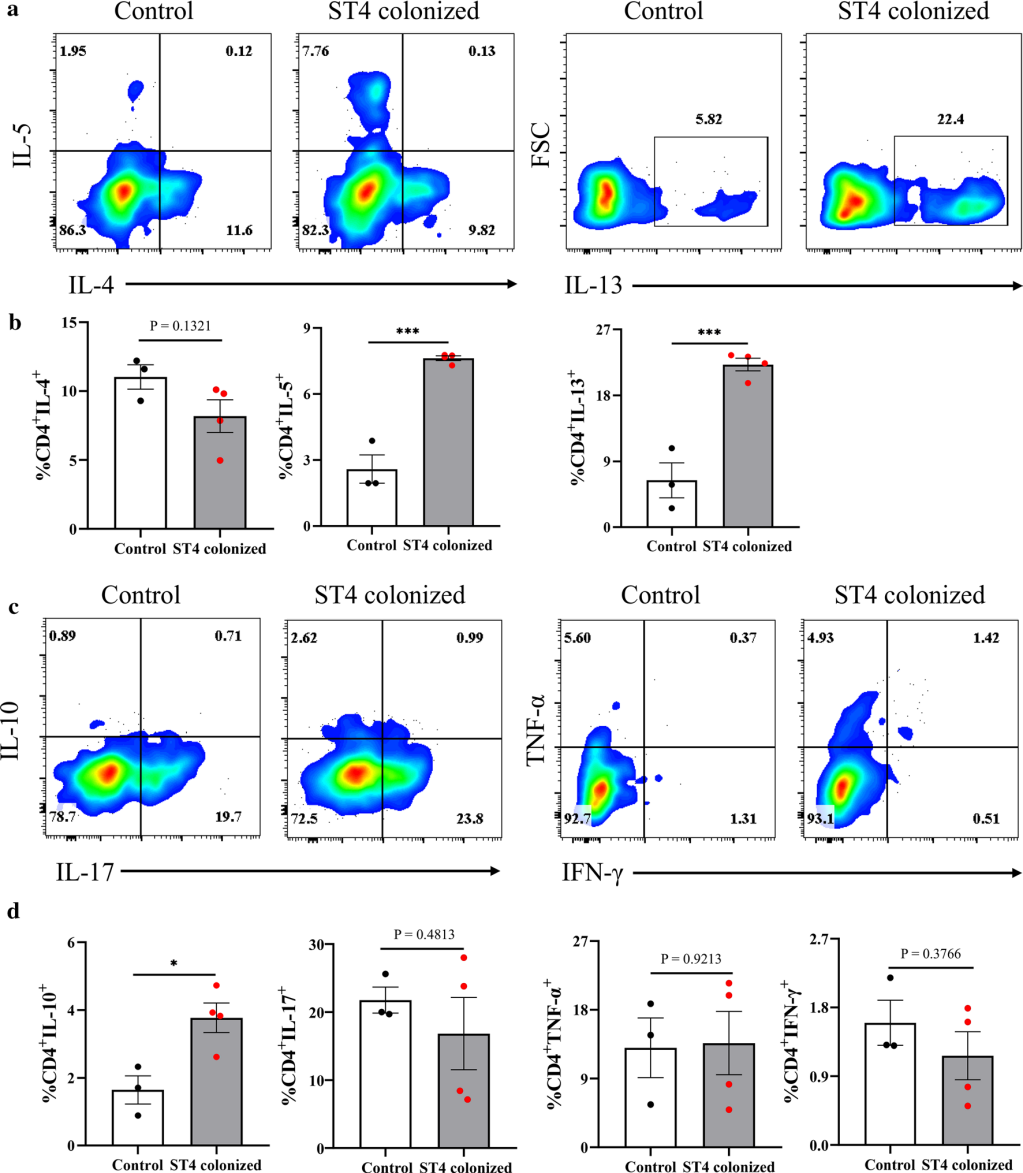
Colonization with *Blastocystis* ST4 induces accumulation of Th2 and Treg cells in the colonic lamina propria (LP). **a** Colored contour plots show staining for IL-4, IL-5, and IL-13 within CD4^+^ cells. **b** Bar charts show the percentage of IL-4, IL-5, and IL-13 expressing CD4^+^ T cells. **c** Colored contour plots show staining for IL-10, IL-17, TNF-α, and IFN-γ within CD4^+^ cells. **d** Bar charts show the percentage of IL-10, IL-17, TNF-α, and IFN-γ expressing CD4^+^ T cells. Statistical significance is indicated by *p < 0.05, and ***p < 0.001, unpaired Student’s *t* test

*Blastocystis* has been reported to involve the host adaptive immune responses. However, these effects are mainly based on in vitro studies [38]. To comprehensively investigate the effect of *Blastocystis* ST4 colonization on adaptive immune responses in a murine model, we examined the polarization status of T cell subsets in the colonic LP from control and ST4-colonized mice (Supplementary Fig. 4). The CD4 T cell population from the *Blastocystis*-associated group was enriched with IL-5-producing cells, reflecting a Th2 differentiated phenotype [39] (Fig. 3a, b). Furthermore, a substantial increase of the Th2 compartment (as defined by the IL-13 expression) was observed in the ST4-colonized group (average 6% and 22% of IL-13-positive cells within the CD4 subset in the control and ST4 group, respectively) (Fig. 3a, b). Notably, together with an increase of the colonic Th2 compartment, the ST4-colonized group displayed a substantial increase in IL-10-producing CD4^+^ Treg cells (Fig. 3c). In contrast, the Th1 compartment (defined by the IFN-γ and TNF-α cytokines) and the Th17 subset (expressing IL-17) were comparable in ST4-colonized mice and control mice (Fig. 3c, d). These data suggest that colonic Th2 and Treg responses are induced by *Blastocystis* ST4 colonization.

### *Blastocystis* ST4 colonizes asymptomatically in Rag1^−/−^ mice

**Fig. 4:**
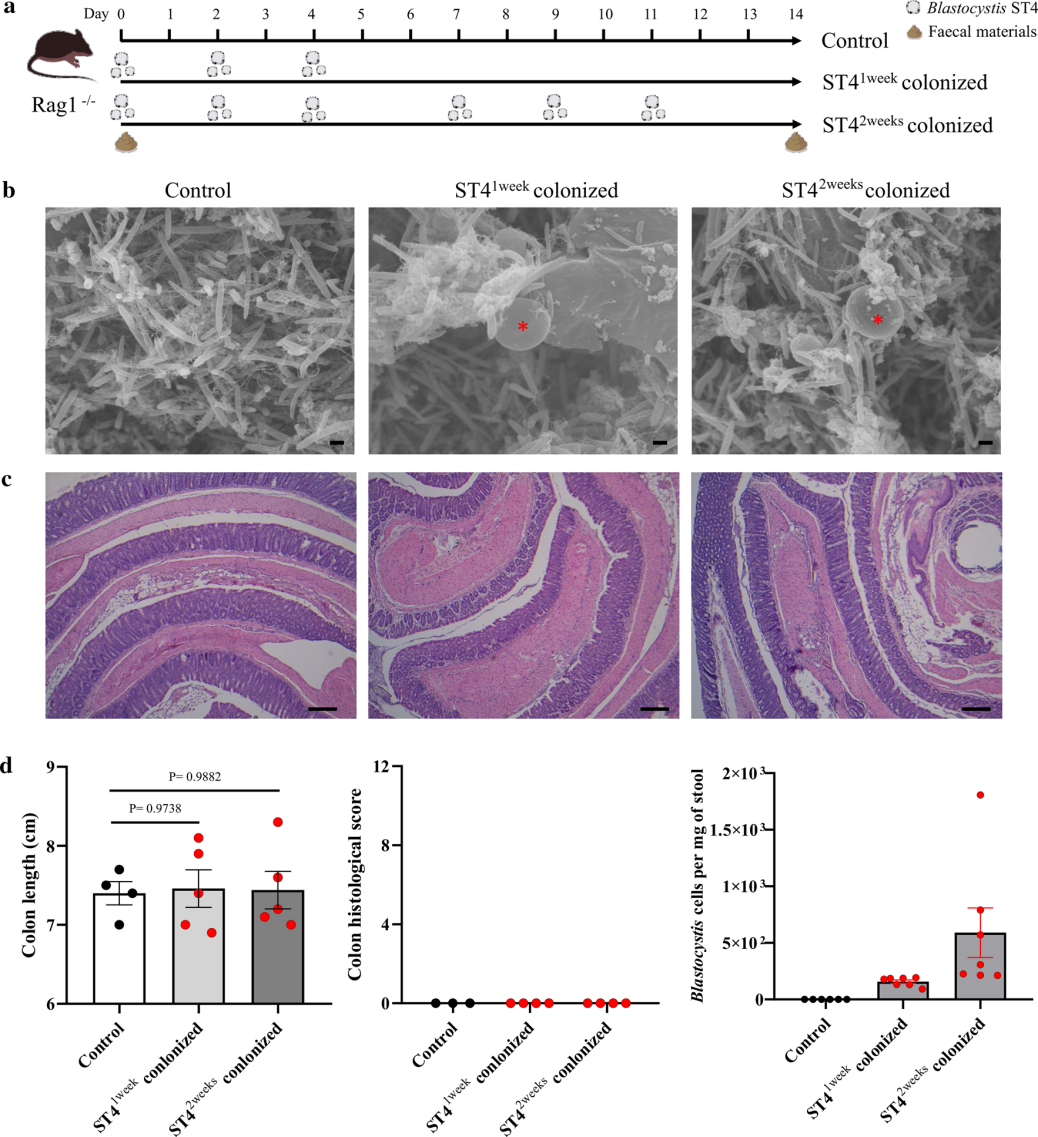
*Blastocystis* ST4 colonizes asymptomatically in *Rag1*^*−/−*^ mice. **a** Experimental design. **b** Scanning electron microscopy (SEM) of colonic and cecum tissues from control and ST4-clonized mice, *Blastocystis* are indicated with red asterisk (*). Scale bar = 1 μm. **c** Representative micrographs of H&E-stained colon sections from ST4-colonized and control mice at day 14. Scale bar = 100 μm. **d** Colon length and colonic histological scores at endpoint. *Blastocystis* ST4 cells per milligram of stool in ST4-clonized mice

Th2 and Treg cells play a crucial role in the host's resistance to parasite infection and in limiting intestinal inflammation [40]. *Blastocystis* ST4 could asymptomatically colonize normal healthy mice, possibly because it activates the host Th2 and Treg cells that promote mucus production, and increases gut motility to maintain intestinal homeostasis [41]. To investigate the role of adaptive immunity in *Blastocystis* ST4 colonization, we carried out experimental infections in immunodeficient *Rag1*^*−/−*^ mice that lack all mature lymphocytes. *Rag1*^*−/−*^ mice were orally inoculated with *Blastocystis* ST4, with extension of the inoculation duration, from 7 to 14 days, to further investigate the effect of *Blastocystis* ST4 colonization in mice (Fig. 4a). Microscopic analysis showed the presence of *Blastocystis* in both ST4^1week^ and ST4^2weeks^ colonized mice (Fig. 4b). Histological examination of the colon tissues revealed no difference in histological scores, and showed intact structure and no inflammatory cell filtrate among the three groups (Fig. 4c, d). Similarly, there was no significant difference in colon length between groups (Fig. 4d). The number of *Blastocystis* cells was determined by qPCR amplification of SSU rRNA gene, and the mice from ST4^2weeks^ colonized group showed higher proportion of *Blastocystis* than ST4^1week^ colonized group (Fig. 4c). These data showed that *Blastocystis* ST4 could asymptomatically colonize *Rag1*^*−/−*^ mice.

### Impact of *Blastocystis* ST4 colonization on the bacterial microbiome of Rag1^−/−^ mice

**Fig. 5:**
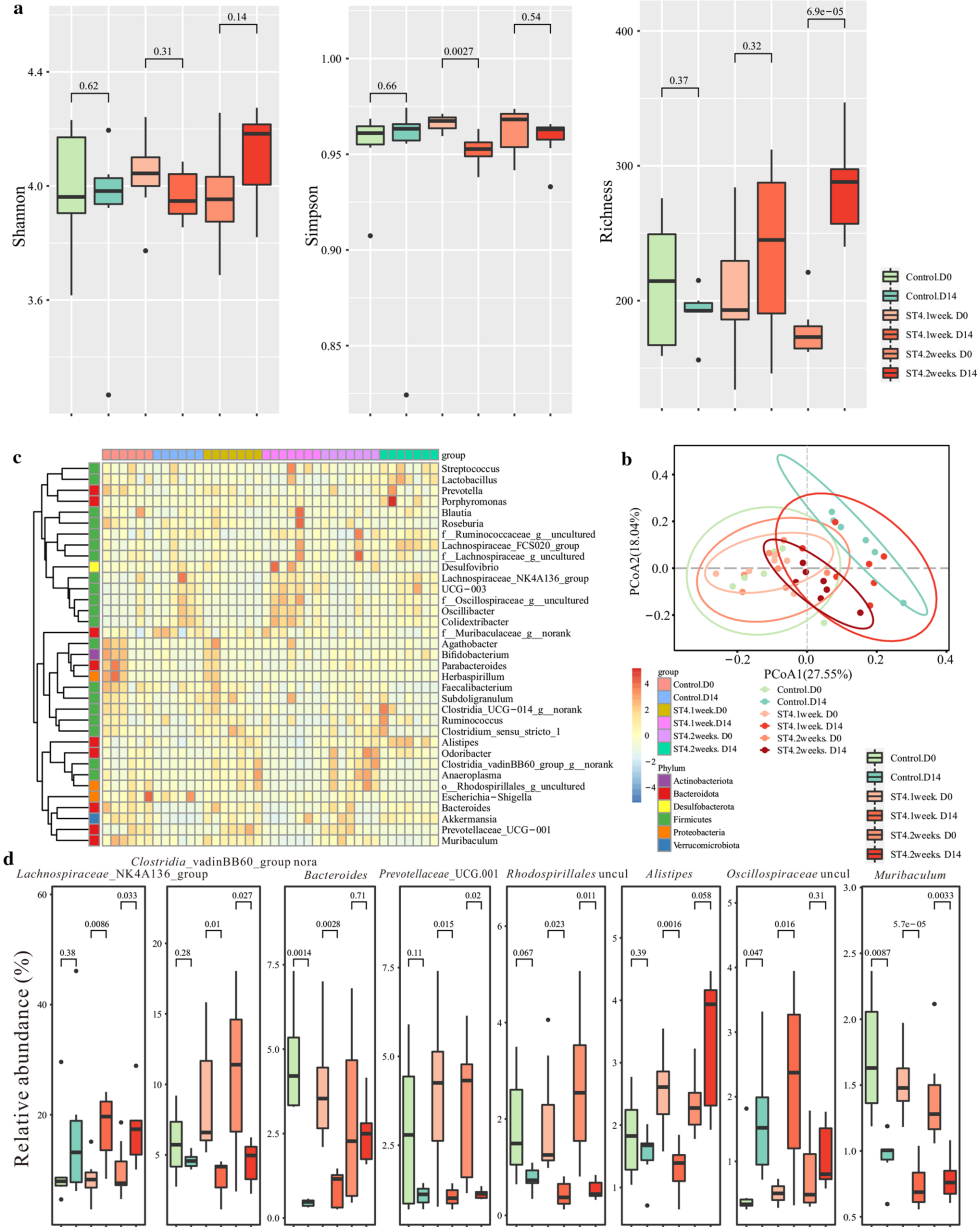
Effect of *Blastocystis* ST4 colonization on fecal bacterial community composition in *Rag1*^*−/−*^ mice. **a** Alpha diversity was measured by Shannon, Simpson, and Richness indexes (*n* = 6 in the control group and *n* = 7 in the ST4-colonized group). **b** PCoA of fecal gut microbiota in control and ST4-clonized mice at day 0 and day 14. **c** Heatmap of ST4 colonization-associated taxonomic markers at day 14. **d** Bacterial genera (relative abundance in the top 35) showing significant differences in their relative abundance between ST4-colonized and control mice

Host adaptive immune responses can regulate the gut microbial compositions [42]. It is unclear whether the changes of intestinal microbiome are caused by the ST4 itself or the interaction of the ST4 and host adaptive immune system. Thus, we further explored the impact of *Blastocystis* ST4 colonization on gut microbial composition in *Rag1*^*−/−*^ mice. Rarefaction curves showed that most of the reads were obtained, thus allowing us to undertake further analysis (Supplementary Fig. 5). There was no significant difference in Shannon index among control, ST4^1week^, and ST4^2weeks^ groups during the experimental period, whereas ST4^1week^ mice revealed decreased Simpson index and ST4^2weeks^ mice increased Richness index at day 14 post-colonization (Fig. 5a). Bacterial community composition changed significantly over time for both ST4-colonized groups (PERMANOVA *p* < 0.01; Fig. 5b; Supplementary Table 1). The relative abundance of various taxa among different groups at day 0 and day 14 are presented as a heatmap (Fig. 5c). Of these bacterial taxa, six genera *Prevotellaceae* UCG.001, unclassified *Clostridia* vadinBB60 group, unclassified *Rhodospirillales*, *Muribaculum*, *Anaeroplasma*, and *Escherichia*–*Shigella* were significantly decreased in the fecal microbiota of both ST4^1week^ and ST4^2weeks^ groups following *Blastocystis* ST4 colonization (Fig. 5d; Supplementary Fig. 6). In contrast, two genera *Lachnospiraceae* NK4A136 group and *Oscillospiraceae* UCG-003 were significantly enriched in ST4-colonizd groups (Fig. 5d; Supplementary Fig. 6). These data collectively suggest that *Blastocystis* ST4 can modulate the gut microbiota in *Rag1*^*−/−*^ mice, even without the participation of B and T immune cells.

### *Blastocystis* ST4 colonization promotes recovery from DSS-induced colitis

**Fig. 6:**
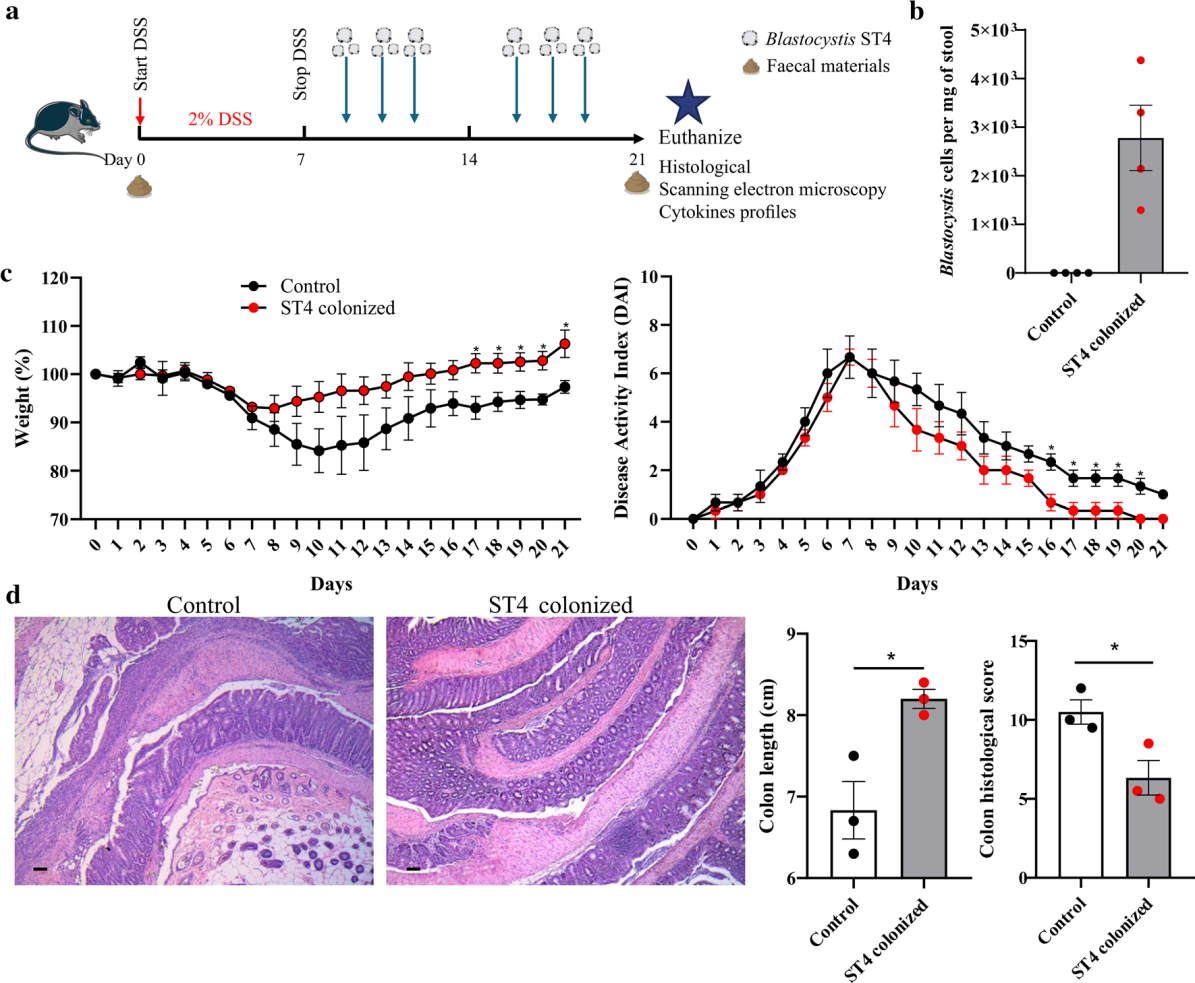
*Blastocystis* ST4 colonization contributes to mice recovery from experimentally induced colitis. **a** Experimental design. **b** *Blastocystis* ST4 cells per milligram of stool in ST4-clonized mice. **c** Weight changes and DAI between control and ST4-colonized mice during treatment. **d** Representative micrographs of H&E-stained colon sections from control and ST4-colonized mice at day 21. Colon length and colonic histological at end point. Scale bar = 100 μm. *p < 0.05, unpaired Student’s *t* test

*Blastocystis* ST7 infection caused colonic tissue damage and ulceration in DSS-induced colitis mice [5], while long-term colonization with *Blastocystis* ST3 promotes a faster recovery from colitis in rats [43], suggesting different subtypes exert differential effects on the host. To further explore whether *Blastocystis* ST4 colonization can modulate the severity of disease in an experiment model of DSS-induced colitis, DSS-treated mice were orally inoculated with *Blastocystis* ST4 three times per week for two consecutive weeks (Fig. 6a, b). Mice with *Blastocystis* ST4 colonization showed faster recovery after DSS-induced colitis, as compared to that in *Blastocystis*-free mice, as reflected by weight changes and DAI (Fig. 6c). Histological examination of the colon revealed reduced mucosal ulceration and damage, and lower histological scores in ST4-clonized mice (Fig. 6d). Similarly, there was significant difference in colon lengths between ST4-colonized mice and control mice (Fig. 6d). These data indicate that *Blastocystis* ST4 colonization promotes recovery of mice from DSS-induced colitis.

### Gut microbiota changes after *Blastocystis* ST4 colonization in DSS-induced colitis mice

**Fig. 7:**
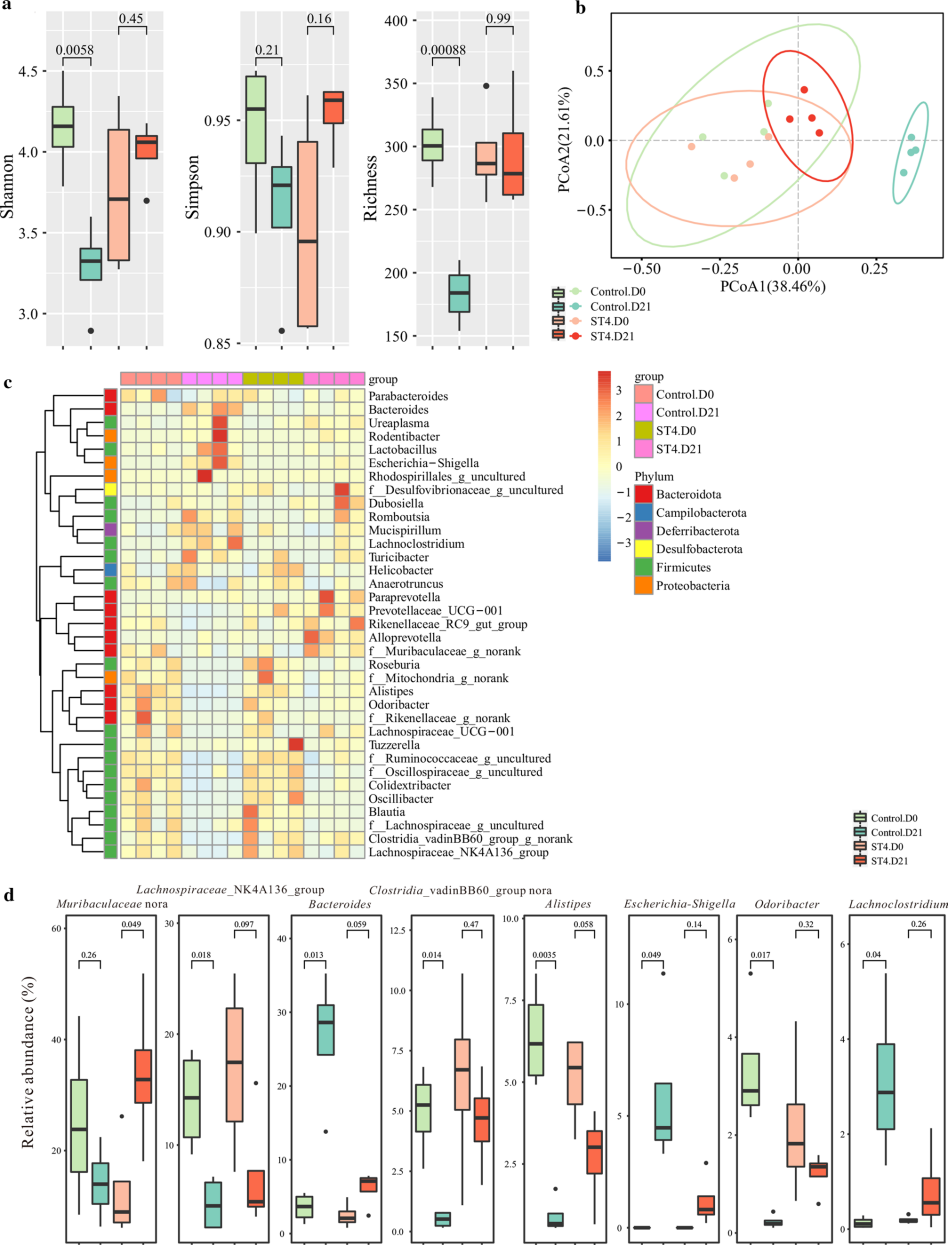
Improvement of experimentally induced colitis is associated with gut microbiota. **a** Alpha diversity was measured by Shannon, Simpson, and Richness indexes (n = 4 per group). **b** PCoA of fecal gut microbiota in control and ST4-clonized mice at day 0 and day 21. **c** Heatmap of ST4 colonization-associated taxonomic markers. **d** Bacterial genera showing significant differences in their relative abundance between ST4-colonized and control mice

Accumulating evidence indicates that the gut microbiome plays a pivotal role in host health and immune homeostasis [44]. The changes in gut microbial composition induced by helminth infection have the ability to reduce intestinal inflammation in an experimental mouse model [45]. To investigate if the protective effect of *Blastocystis* ST4 on DSS-induced colitis mice is related to alterations in the gut bacterial community, we compared the variations in the microbial communities in ST4-colonized and control mice over time. Reduced alpha diversity (Shannon and Richness indexes) was observed in control mice, whereas *Blastocytsis* ST4 colonization maintained a stable alpha diversity over time (Supplementary Fig. 7; Fig. 7a). Both ST4-colonized and control group showed pronounced differences in bacterial composition during the experiment period (PERMANOVA *p* < 0.05; Fig. 7b; Supplementary Table 1). The relative abundance of various taxa are shown as a heatmap among groups (Fig. 7c). In particular, the genera *Lachnospiraceae* NK4A136 group, unclassified *Clostridia vadinBB60* group, *Alistipes*, *Odribacter*, *Oscillibacter*, *Colidextribacter*, unclassified *Oscillospiraceae*, unclassified *Ruminococcaceae*, *Roseburia*, and uncultured *Mitochondria* were significantly reduced in control mice over experimental time, whereas the genera *Bacteroides*, *Escherichia–Shigella*, *Lachnoclostridium*, and *Paraprevotella* were progressively expanded in control mice over time (Fig. 7d; Supplementary Fig. 8). Conversely, colonization with *Blastocystis* ST4 appears to maintain a relatively stable microbiota communities compared to *Blastocystis*-free mice after DSS administration. Higher proportions of unclassified *Muribaculaceae* and lower abundance of *Oscillibacter*, *Colidextribacter*, unclassified *Oscillospiraceae* and unclassified *Ruminococcaceae* were observed in ST4-colonized mice over time (Fig. 7d, Supplementary Fig. 8). Overall, these results suggest that the protective effect of *Blastocystis* ST4 on DSS-induced colitis mice was associated with a stable bacterial diversity and microbiota communities.

### Protective effects of *Blastocystis* ST4 in DSS-induced colitis mice may be mediated by activation of Th2 and Treg cells responses

**Fig. 8:**
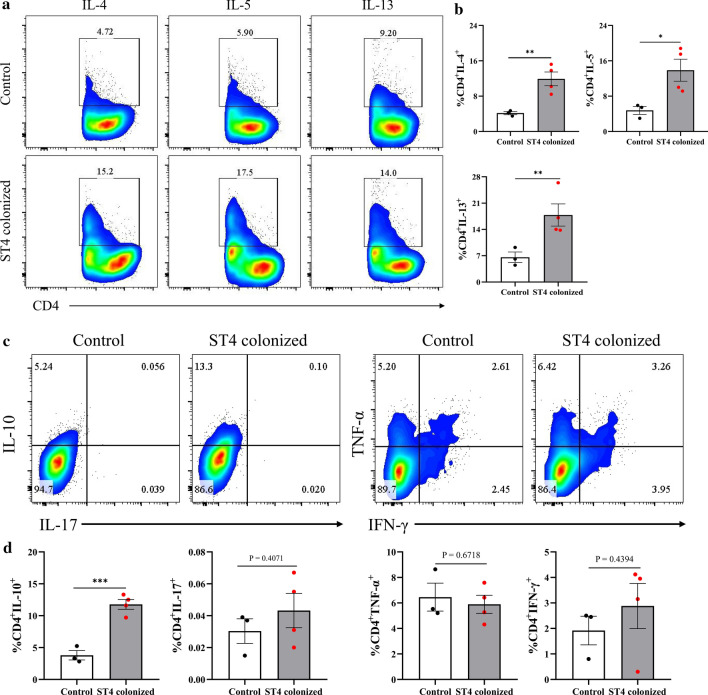
*Blastocystis* ST4 colonization activates Th2 and Treg cells responses in colonic mucosa of DSS-induced colitis mice. **a** Colored contour plots show staining for IL-4, IL-5, and IL-13 within CD4^+^ cells. **b** Bar charts show the percentage of IL-4, IL-5, and IL-13 expressing CD4^+^ T cells. **c** Colored contour plots show staining for IL-10, IL-17, TNF-α, and IFN-γ within CD4^+^ cells. **d** Bar charts show the percentage of IL-10, IL-17, TNF-α, and IFN-γ expressing CD4^+^ T cells. Statistical significance is indicated by *p < 0.05, **p < 0.01, and ***p < 0.001, unpaired Student’s *t* test

Helminth-mediated Th2 or Treg cell responses have been exploited to ameliorate experimental colitis in a mouse model [46, 47]. We asked if *Blastocystis* ST4 also regulates these immune responses to confer protection from DSS-induced colitis. Colonic tissues from DSS-induced colitis mice with *Blastocystis* ST4 colonization demonstrated increased expression of Th2 (IL-4, IL-5, and IL-13) cytokines relative to control mice (Fig. 8a, b). The immunophenotype of ST4-colonized DSS-treated mice was consistent with the previous observations on normal mice (Fig. 3a, b). Importantly, the size of the anti-inflammatory cytokine IL-10-producing Treg cell subset in colonic LP was also elevated in ST4-colonized mice (Fig. 8c, d). Similar to what we observed in normal healthy mice, the numbers of Th1-asscociated cytokine expressing cells (IFN-γ^+^ and TNF-α^+^), as wells as Th17 cells (IL-17) were similar in ST4-colonized and control mice (Fig. 8c, d). Collectively, the composition of the *Blastocystis*-associated CD4 compartments in colonic LP from the DSS-induced colitis model indicated this organism's direct interaction with the host’s adaptive immune system. Furthermore, *Blastocystis*-driven changes in the gut microbiome with the accompanying shift within immune compartments are plausible key factors that account for attenuation in the severity of DSS-mediated colitis.

### Transfer of fecal microbiota from ST4-colonized Rag1^−/−^ mice reduces inflammation in experiment-induced colitis

**Fig. 9:**
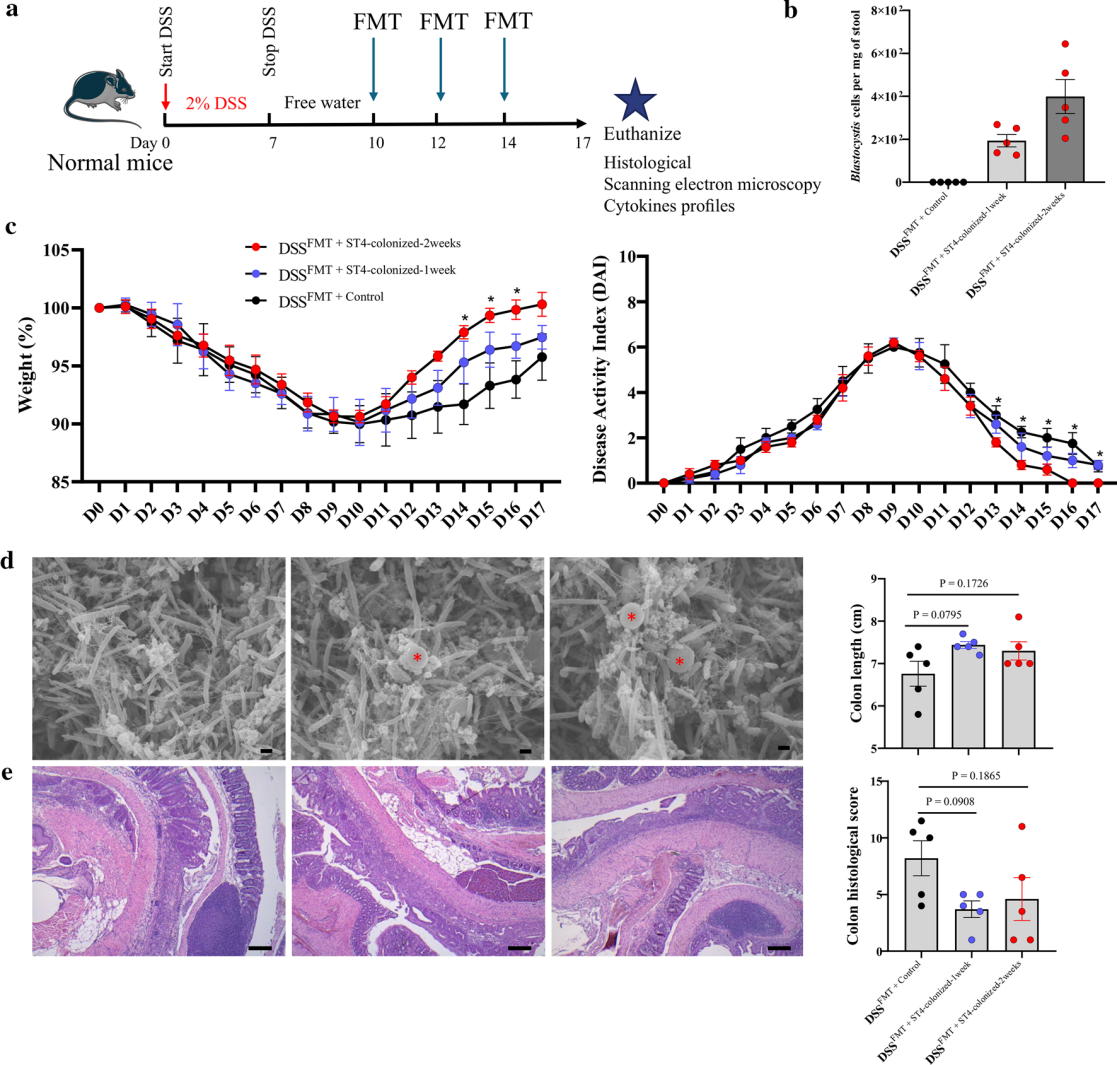
Transfer of ST4-altered microbiome to colitis mice reduces colonic inflammation. **a** Experimental design. **b** *Blastocystis* ST4 cells per milligram of stool in DSS^FMT + ST4−clonized^ mice. **c** Weight changes and DAI between DSS^FMT + Control^ mice and DSS^FMT + ST4−clonized^ mice. **d** Scanning electron microscopy (SEM) of colonic and cecum tissues from DSS^FMT + Control^ mice and DSS^FMT + ST4−clonized^ mice, *Blastocystis* are indicated with red asterisk (*). Scale bar = 1 μm. **e** Representative micrographs of H&E-stained colon sections from DSS^FMT + Control^ mice and DSS^FMT + ST4−clonized^ mice at day 17. Scale bar = 100 μm. Colon length and colonic histological scores at end point

**Fig. 10:**
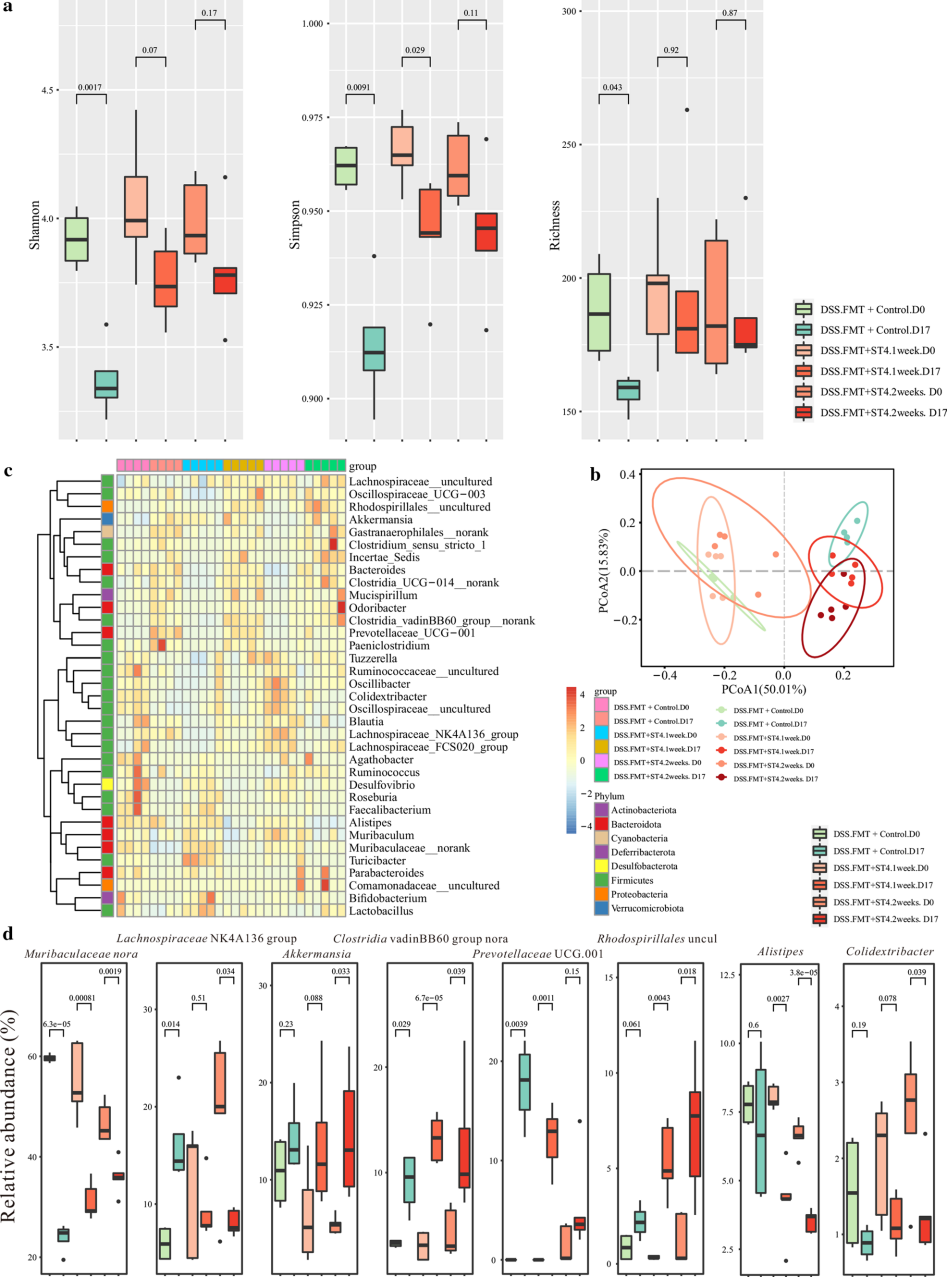
Gut microbiota analysis upon different FMT conditions treatment in DSS-induced colitis mice. **a** Alpha diversity was measured by Shannon, Simpson, and Richness indexes (n = 4 in control group and n = 5 in ST4-colonized group). **b** PCoA of fecal gut microbiota in control and ST4-clonized mice at day 0 and day 17. **c** Heatmap of taxonomic markers among different groups. **d** Bacterial genera showing significant differences in their relative abundance among groups

Next, to determine the effect of *Blastocystis* ST4-altered microbial communities that was independent of adaptive immunity-mediated microbiota changes on experiment-induced colitis, we performed FMT from ST4-colonized *Rag1*^*−/−*^ mice into DSS-treated mice (Fig. 9a). DSS^FMT + ST4−colonized^ mice showed faster recovery and better health status, as measured by weight changes and DAI (Fig. 9c), compared to DSS^FMT + control^. In addition, lower levels of intestinal inflammation and higher colon length were detected in DSS^FMT + ST4−colonized^ mice, although did not reach significance difference (Fig. 9e). We further evaluated the bacterial communities in experiment-induced mice upon different FMT conditions. Rarefaction curves showed that the depth of sequencing was sufficient for analysis (Supplementary Fig. 9). DSS^FMT+ST4−colonized^ mice showed a stable bacterial α-diversity when compared to DSS^FMT+Control^ mice, which showed significant decreases, as indicated by the Shannon, Simpson and Richness indices (Fig. 10a). The gut microbial communities changed significantly over time for DSS^FMT + ST4−colonized^ and DSS^FMT+Control^ groups (PERMANOVA *p* < 0.05; Fig. 10b; Supplementary Table 1). The differences in the relative abundance of various taxa were observed in heatmap format (Fig. 10c). We observed higher levels of commensals and SCFA-producing taxa, *Akkermansia*, unclassified *Clostridia* vadinBB60 group, uncultured *Rhodospirillales*, and *Clostridia* UCG.014 in DSS^FMT + ST4−colonized^ mice (Fig. 10d; Supplementary Fig. 10). DSS^FMT + Control^ mice showed the enrichment of the genera *Lachnospiraceae* NK4A136 group, unclassified *Clostridia* vadinBB60 group, *Prevotellaceae* UCG.001, *Bacteroides*, *Blautia*, and *Clostridia* UCG.014 (Fig. 10d; Supplementary Fig. 10). Altogether, these findings suggest that transfer of ST4-altered gut microbiome are beneficial to the recovery of experimental colitis.

### FMT influences SCFAs and Treg cells IL-10 production in DSS-induced colitis recipients

**Fig. 11:**
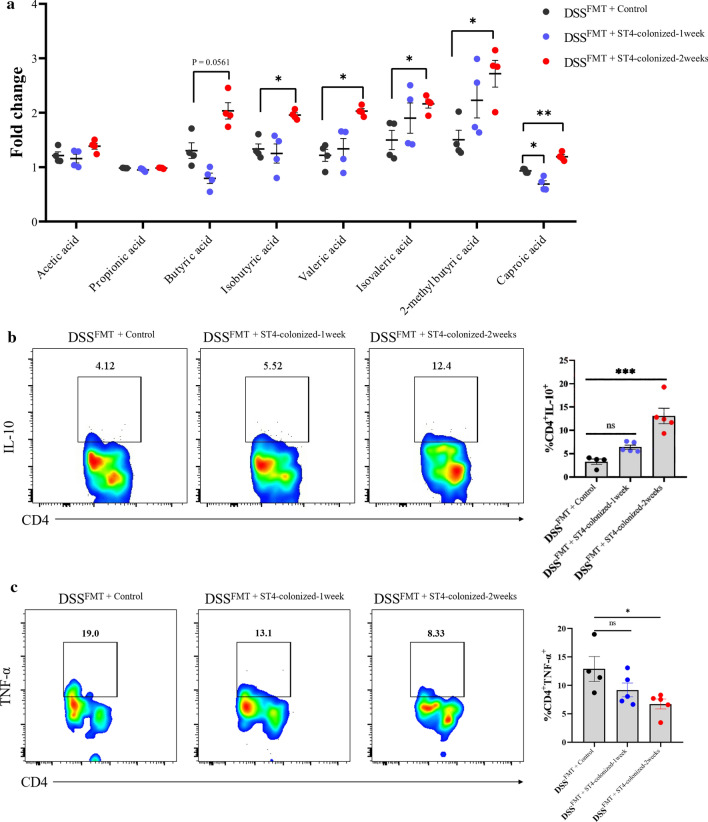
FMT influences SCFAs and Treg cells IL-10 production in DSS-induced colitis recipients. **a** Fold change of each SCFA relative to levels at day 0 from DSS^FMT^ mice (recipients). **b** Colored contour plots show staining for IL-10 within CD4^+^ cells, and bar chart shows the percentage of IL-10 expressing CD4^+^ T cells. **c** Colored contour plots show staining for TNF-α within CD4^+^ cells, and bar chart shows the percentage of TNF-α expressing CD4^+^ T cells. *p < 0.05, **p < 0.01, ***p < 0.001, Two-way ANOVA (**a**) and one-way ANOVA (**b, c**) analysis with Tukey multiple comparison test

The host microbiome plays an important role in regulating physiology through microbiota-derived metabolites, especially SCFAs, during host–microbiome interactions [48]. To gain mechanistic insight into the faster recovery from intestinal inflammation in DSS^FMT +ST4−colonized^ mice, we firstly quantitated eight SCFAs (acetic, propionic, butyric, isobutyric, valeric acid, isovaleric, 2-methylbutyric, and caproic acid) of feces by LC/MS/MS. The levels of five SCFAs in fecal samples of DSS^FMT +ST4−colonized−2 weeks^ mice exhibited significantly higher concentration changes than DSS^FMT +control^ mice (Fig. 11a). SCFAs are critical to the immune system and can serve as substrates for host energy metabolism [49]. We also detected increased SCFAs in *Rag1*^*−/−*^ mice after *Blastocystis* ST4 colonization for one or two weeks (Supplementary Fig. 11). Besides, it has been determined that microbiota-derived SCFAs have the ability to modulate Treg cell function and can alleviate the colonic inflammation [50, 51]. We then assessed whether re-colonization of DSS treated mice with microbiota from ST4-colonized mice via FMT influences colonic immune cells. Interestingly, we observed an increased number of CD4^+^ cells expressing IL-10 and decreased number of CD4^+^ cells expressing TNF-α in DSS^FMT + ST4−colonized^ mice (Fig. 11b, c). Taken together, these data indicate that transfer of ST4-altered gut microbiota in DSS-induced colitis recipients increases SCFAs production and induces accumulation of IL-10-producing Treg cells.

## Discussion

Although the pathogenicity of *Blastocystis* is controversial, accumulating evidence shows that it is often present in asymptomatic individuals and is associated with healthy gut microbiota [52]. To understand the causal role of *Blastocystis* ST4 on the host gut microbiota and mucosal immune responses, we established murine models of oral colonization to investigate the effects of *Blastocystis* colonization on gut microbiota, and adaptive immune responses. Our results suggest that *Blastocystis* ST4 colonization in normal healthy and *Rag1*^*−/−*^ mice did not cause any pathological lesions or inflammatory cell infiltration in colonic mucosa. Furthermore, *Blastocystis* ST4 colonization and transfer of ST4 colonization-altered gut microbiota to experimentally induced colitis mice promoted faster recovery from experimental caused colitis.

Microbial alpha diversity is considered an important marker for gut health, and high bacterial diversity implies stability and resilience of the gut ecosystem [53]. We monitored changes in the alpha diversity over the course of colonization with *Blastocystis* ST4 in several mouse models. Interestingly, alpha diversity (measured by Richness index) was significantly increased in *Rag1*^*−/−*^ mice colonization with *Blastocystis* ST4 at day 14 compared with day 0 (Fig. 5a). Similarly, although it's a different model of infection, a previous study showed infection with ST4 cysts increase in bacterial richness in rats [36]. The higher bacterial richness was also observed in individuals with *Blastocystis* colonization in the majority of microbiome studies [54, 55]. On the other hand, colonization with ST4 appears to maintain a stable fecal bacterial alpha diversity in normal healthy and DSS-induced colitis mice, while a significant decrease in alpha diversity was detected in control mice after DSS administration (Figs. 2a, 7a, and 10a). Loss of alpha diversity has been implicated in IBD patients [56, 57], and it is also a sign of dysbiosis in many other human diseases [58]. Thus, the decreased alpha diversity in control mice but not ST4-colonized mice may explain the differences in recovery from colitis.

*Blastocystis* and gut microbiota co-inhabit the host intestinal tract and are capable of interacting with each other. We observed that ST4 colonization significantly changed the bacterial community compositions, but there were some differences in specific taxa, depending on the mouse model utilized. For example, ST4 colonization mainly increased the abundance of *Clostridia vadinBB60* group, belonging to the *Clostridia* class, in normal healthy mice, which was positively correlated with the Treg cell counts [59]. *Lachnospiraceae* NK4A136 group, one of the known short-chain fatty acid (SCFA)-producing bacteria that degrade complex polysaccharides [60], was the most enriched bacteria in ST4-colonized *Rag1*^*−/−*^ mice. Hu et al. reported that the elevated abundance of *Lachnospiraceae* NK4A136 group showed anti-inflammation effects in obese mice [61]. The initial abundances of *Clostridia vadinBB60* group and *Lachnospiraceae* NK4A136 group across different mouse models were different, which may have contributed to the inconsistent results across different mouse models (Figs. 2d, 5d). Furthermore, host adaptive immune responses can also regulate gut microbial compositions [42]. In addition to the direct effect of *Blastocystis* ST4 on the gut microbiota in normal healthy mice and *Rag1*^−/−^ mice (which lack mature lymphocytes), the host's adaptive immune response would have influenced the composition of gut microbiota, as evidenced in the day 0 data.

Notably, we observed *Blastocystis* ST4 colonization in DSS-induced colitis inhibits the expansion of *Bacteroides* and *Escherichia*–*Shigella*. It has been determined that inhibition of expansion of *Bacteroides* can alleviate intestinal inflammation in *Nod2*^−/−^ mice [62]. *Escherichia*–*Shigella*, belonging to the family *Enterobacteriaceae*, are one of the most important enteric pathogens causing gastroenteritis worldwide [63]. A previous study reported that the expansion of *Escherichia*–*Shigella* was associated with lower bacterial diversity and pro-inflammatory effects [64]. Therefore, our study demonstrated that the improvement of intestinal inflammation by *Blastocystis* ST4 colonization may be related to the inhibition of these pathobiont bacteria.

FMT has been successfully used in the treatment of *Clostridium difficile* infection (CDI) and IBD [65, 66]. An interesting study included *Blastocystis*-positive (ST1 and ST3) donor samples for FMT treatment of recurrent CDI, and demonstrated that the presence of *Blastocystis* ST1 and ST3 from donors did not cause any adverse gastrointestinal symptoms [67]. In our study, we found that transfer ST4-altered microbiota from *Rag1*^−/−^ mice reduces inflammation in experiment-induced colitis through an increase in “beneficial” microbes such as *Akkermansia*. Bacteria belonging to *Akkermansia* are associated with gut health, and the expansion of *Akkermansia* can increase mucus production to ameliorate intestinal inflammation [68]. Moreover, FMT from ST4-colonized mice increased SCFAs production and the proportion of anti-inflammatory cytokine IL-10 more profoundly than FMT from control mice. This is the first study to demonstrate that FMT from a donor colonized with *Blastocystis* ST4 improves the intestinal inflammation in a mouse model. Although we did not dissociate the effect of ST4 together with altered microbiota from the effect of the ST4 itself during the FMT, we determined that the beneficial effects of ST4 on the host are transferable.

The gut microbial community plays an important role in the development and modulation of the immune system [69]. We observed that *Blastocystis* ST4 colonization activates Th2 immune responses in normal healthy mice and DSS-induced colitis mice. It has been determined that Th2 cells are important sources of type 2 cytokines (IL-4, IL-5 and IL-13) and are also important effector cells during the inflammatory process [70]. Recent data showed an expansion of IL-13- and IL-4-producing CD4^+^ T cells in *Nod2*^−/−^ mice contributes to ameliorating the intestinal injury response [62]. Another interesting study in primates demonstrated that the experimental administration of *Trichuris trichiura* can ameliorate colitis by both induction the colonic CD4^+^ T cells producing IL-4 and modulation of microbial populations [71].

In addition, we also found that *Blastocystis* ST4 colonization increases the number of IL-10-producing Treg in the colonic mucosa of DSS-induced mice. The cytokine IL-10 produced by Treg cells is required for containment of inflammatory responses in mucosal tissues [72]. Both humans and mice deficient in IL-10 or IL-10 receptor (IL-10R) are prone to develop severe intestinal inflammation [73–75], highlighting the importance of IL-10 in preventing this disease process. On the other hand, the gut microbe-derived SCFAs can enhance the expression of Foxp3 and IL-10-expressing colonic Tregs by inhibition of histone deacetylase (HDAC) or in a GPR43-dependent manner [50, 76, 77]. Although the three most abundant SCFAs, acetate, propionate, and butyrate, did not change significantly after FMT and ST4 colonization, there was elevation in the other five SCFAs, isobutyric acid, valeric acid, isovaleric acid, 2-methylbutyric acid, and caproic acid, which have been reported to induce the accumulation of anti-inflammatory cytokine IL-10 [78–80]. However, it is unclear whether the increased production of IL-10 is directly caused by *Blastocystis* ST4 or indirectly through regulating the gut microbiota-derived SCFAs. Future studies should focus on understanding the mechanistic connection between *Blastocystis* ST4 colonization, IL-10 signaling, and bacterial-derived SCFAs using relevant animal models.

## Conclusions

We demonstrated that *Blastocystis* ST4 colonization is able to alter the gut bacterial community composition in an adaptive immunity-independent fashion, evidenced through the use of *Rag1*^*−/−*^ mice. In models with intact immune systems, *Blastocystis* ST4 induces the expression of Th2 and Treg cytokines. Notably, alterations in gut microbiota composition mediated by *Blastocystis* ST4 colonization is associated with amelioration of colonic inflammation, likely through immunomodulatory effects of SCFAs, Th2 and Treg effectors. These findings represent an important contribution toward the elucidation of the complex interplay between *Blastocystis* ST4, gut microbiota, and host adaptive immune responses.

### Supplementary Information

Below is the link to the electronic supplementary material.

Supplementary file1 (TIF 8999 KB)** Figure S1. ***Blastocystis* ST4 colonization did not induce any abnormal effects on C57BL/6 mice. **a**, Representative micrographs of H&E-stained caecum sections and histological scores from ST4-colonized and control mice at day 7. **b**, Representative micrographs of H&E-stained small intestine sections and histological scores from ST4-colonized and control mice at day 7. Scale bar = 100 μm.

Supplementary file2 (TIF 482 KB)** Figure S2.** Rarefaction curves (threshold is 90,000) showing microbial diversity based on the Shannon index (upper panel) and Observed features (bottom panel) from normal healthy mice.

Supplementary file3 (TIF 184 KB)** Figure S3. **Comparison of relative abundancies of different taxa between control and ST4-colonized mice.

Supplementary file4 (TIF 9690 KB)** Figure S4.** Gating strategy of the immune compartments isolated from colonic lamina propria.

Supplementary file5 (TIF 580 KB)** Figure S5.** Rarefaction curves (threshold is 24,000) showing microbial diversity based on the Shannon index (upper panel) and Observed features (bottom panel) from *Rag1*^*−*^^*/*^^*−*^ mice.

Supplementary file6 (TIF 311 KB)** Figure S6.** Comparison of relative abundancies of different taxa between control and ST4-colonized mice.

Supplementary file7 (TIF 526 KB)** Figure S7.** Rarefaction curves (threshold is 58,000) showing microbial diversity based on the Shannon index (upper panel) and Observed features (bottom panel) from DSS-induced colitis mice.

Supplementary file8 (TIF 236 KB)** Figure S8. **Comparison of relative abundancies of different taxa between control and ST4-colonized mice.

Supplementary file9 (TIF 826 KB)** Figure S9. **Rarefaction curves (threshold is 26,000) showing microbial diversity based on the Shannon index (upper panel) and Observed features (bottom panel) from DSS^FMT^ mice.

Supplementary file10 (TIF 290 KB)** Figure S10. **Comparison of relative abundancies of different taxa between control and ST4-colonized mice.

Supplementary file11 (TIF 1457 KB)** Figure S11. **Fold-change of each SCFA relative to levels at day 0 from *Rag1*^*-/-*^ mice (donor mice).

Supplementary file12 (XLSX 10 KB)** Table S1.** PERMANOVA of beta-diversity analysis as measured by Bray-Curtis dissimilarity.

Supplementary file13 (XLSX 620 KB)** Table S2.** Sample metadata, feature table and taxonomic classifications.

## Data Availability

The datasets generated and analyzed in the current study are available in the Sequence Read Archive (SRA) database at NCBI under BioProject ID PRJNA669121 (https://www.ncbi.nlm.nih.gov/bioproject/PRJNA669121).

## Abbreviations

IBD: Inflammatory bowel disease
IBS: Irritable bowel syndrome
SSU: Small subunit
Th2: T helper 2
Treg: T regulatory
FGFP: Flemish Gut Flora Project
AGP: American Gut Project
IMDM: Iscove’s modified Dulbecco’s medium
PBS: Phosphate-buffered saline
DSS: Dextran sulfate sodium
DAI: Disease activity index
H&E: Hematoxylin and eosin
SI: Small intestine
SEM: Scanning electron microscopy
QIIME: Quantitative Insights into Microbial Ecology
RPMI: Roswell Park Memorial Institute
FCS: Fetal calf serum
FBS: Fetal bovine serum
IL: Interleukin
IFN-γ: Interferon gamma
TNF-α: Tumor necrosis factor alpha
LP: Lamina propria
SCFAs: Short-chain fatty acids
CDI: *Clostridium difficile* Infection
CD: Crohn’s disease
HDAC: Histone deacetylase
