# Supplementary figures and images for: Experimental colonization with Blastocystis ST4 is associated with protective immune responses and modulation of gut microbiome in a DSS-induced colitis mouse model
Source: Cell Mol Life Sci. 2022 Apr 18;79(5):245. doi: 10.1007/s00018-022-04271-9 (PMC9016058; doi:10.1007/s00018-022-04271-9)

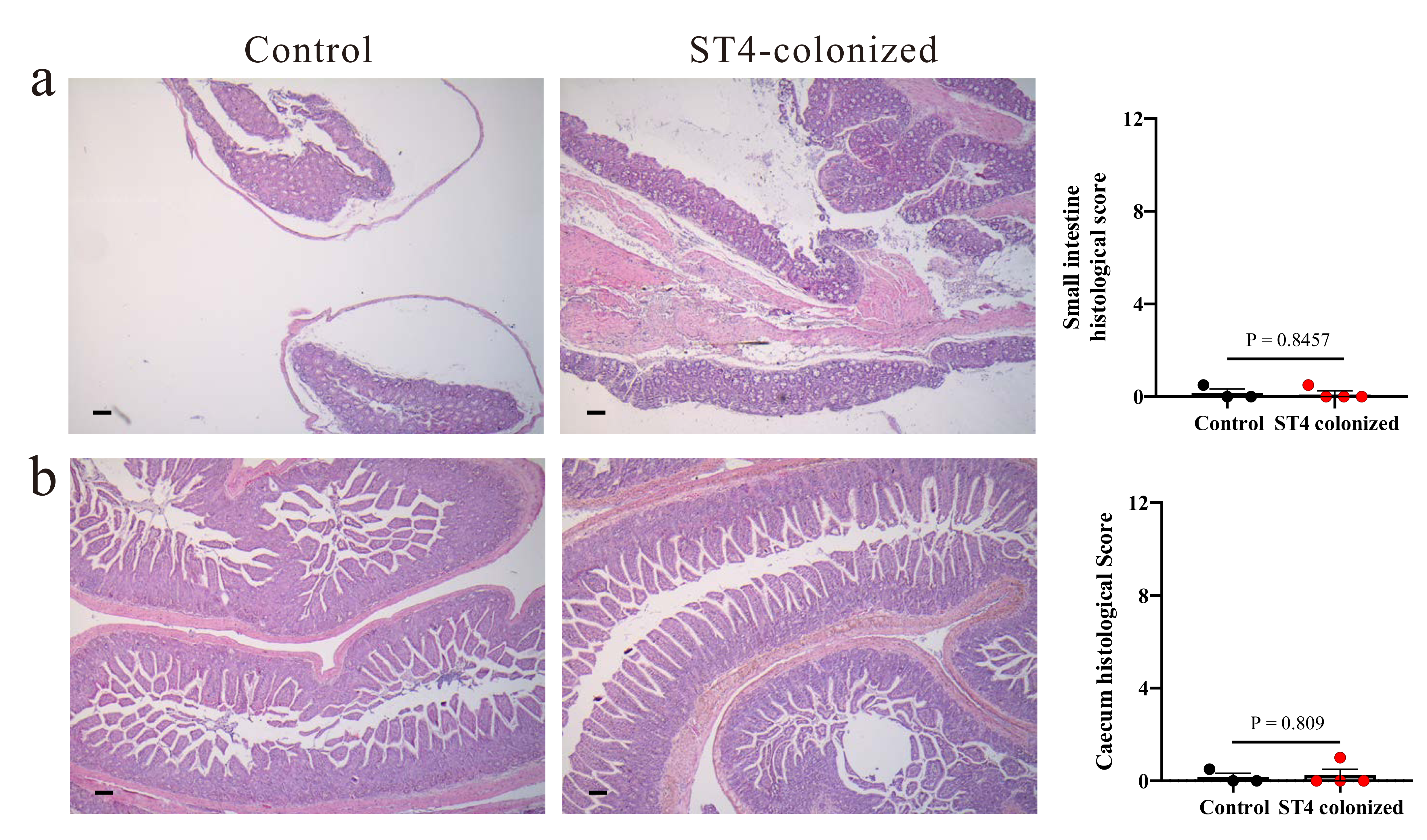

Supplement: Supplementary file 1 — Supplementary file1 (TIF 8999 KB) Figure S1. Blastocystis ST4 colonization did not induce any abnormal effects on C57BL/6 mice. a, Representative micrographs of H&E-stained caecum sections and histological scores from ST4-colonized and control mice at day 7. b, Representative micrographs of H&E-stained small intestine sections and histological scores from ST4-colonized and control mice at day 7. Scale bar = 100 μm. [file 18_2022_4271_MOESM1_ESM.tif]

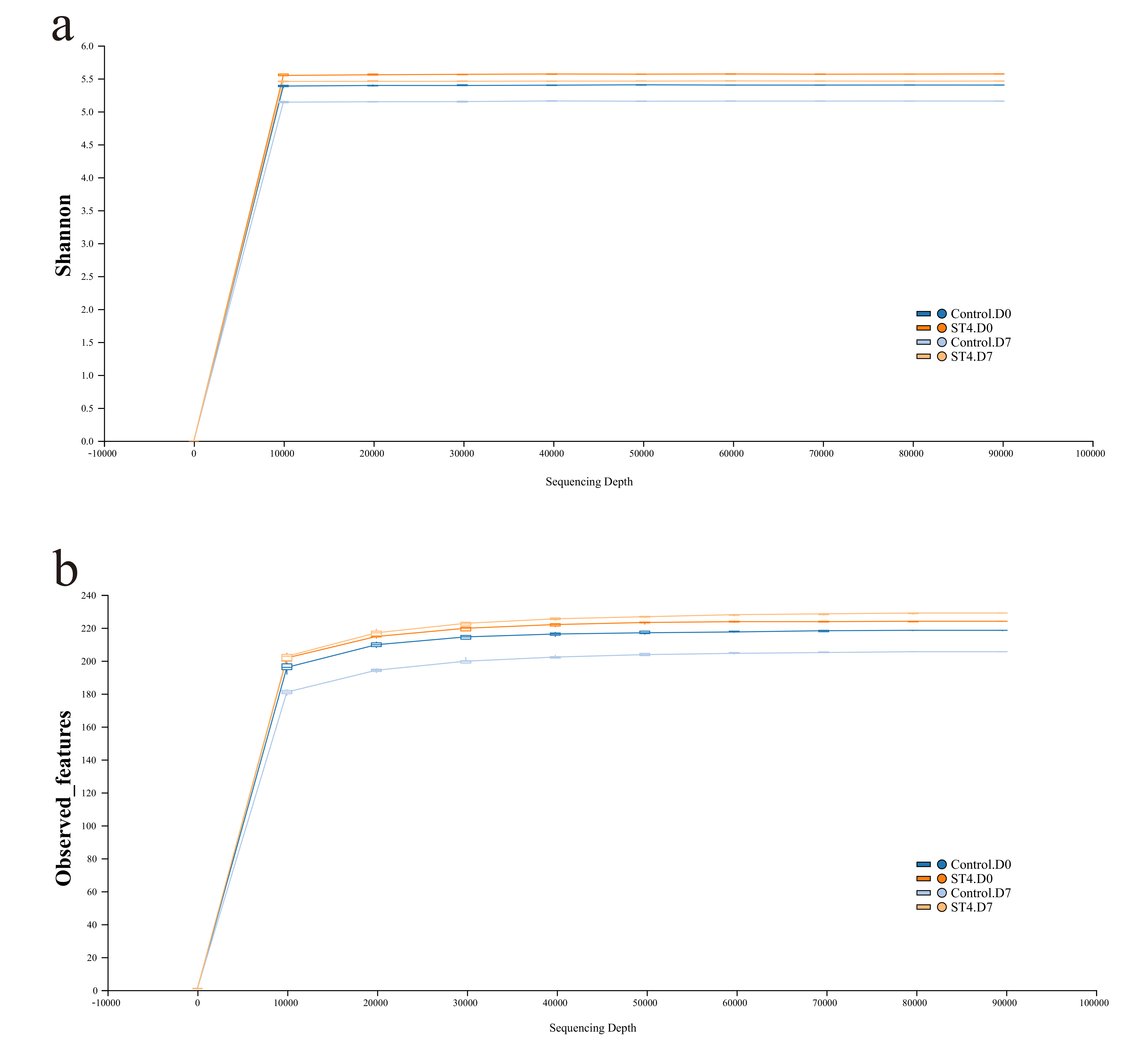

Supplement: Supplementary file 2 — Supplementary file2 (TIF 482 KB) Figure S2. Rarefaction curves (threshold is 90,000) showing microbial diversity based on the Shannon index (upper panel) and Observed features (bottom panel) from normal healthy mice. [file 18_2022_4271_MOESM2_ESM.tif]

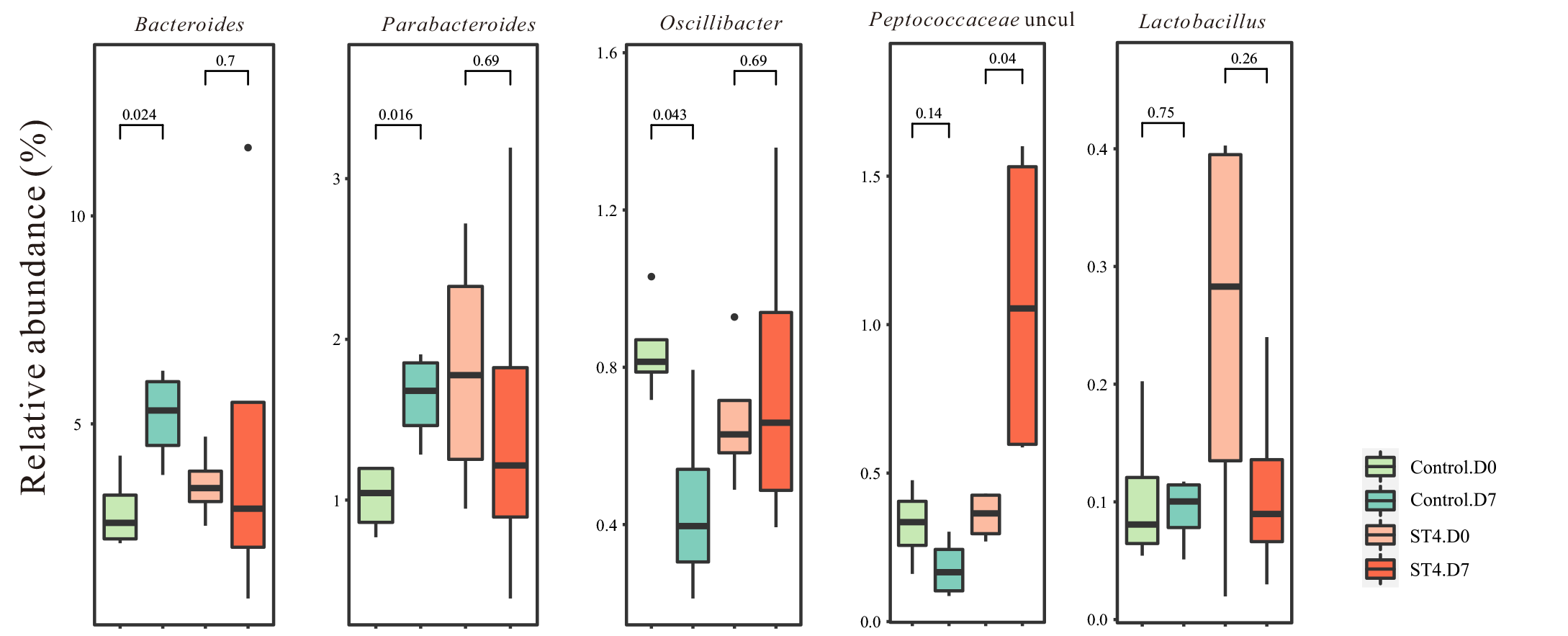

Supplement: Supplementary file 3 — Supplementary file3 (TIF 184 KB) Figure S3. Comparison of relative abundancies of different taxa between control and ST4-colonized mice. [file 18_2022_4271_MOESM3_ESM.tif]

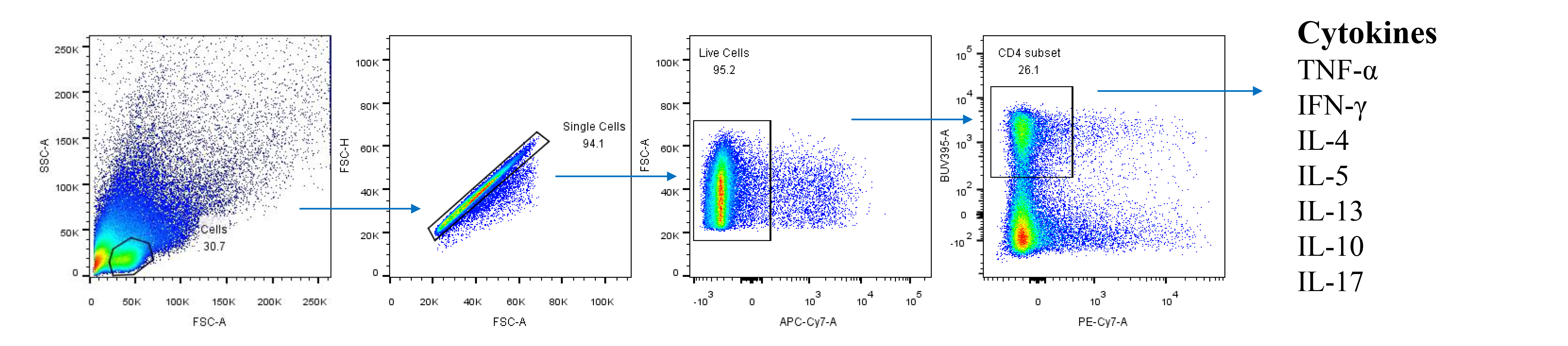

Supplement: Supplementary file 4 — Supplementary file4 (TIF 9690 KB) Figure S4. Gating strategy of the immune compartments isolated from colonic lamina propria. [file 18_2022_4271_MOESM4_ESM.tif]

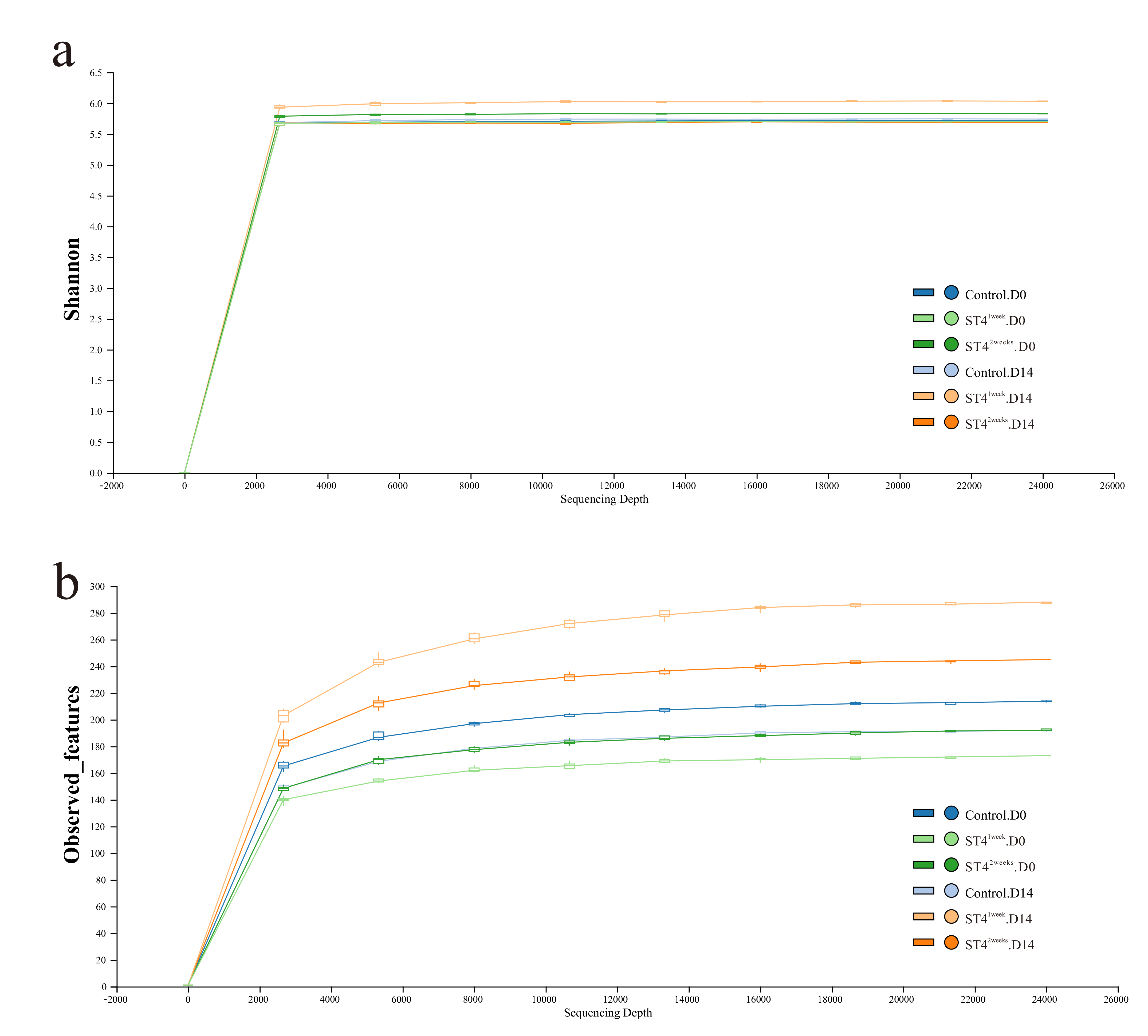

Supplement: Supplementary file 5 — Supplementary file5 (TIF 580 KB) Figure S5. Rarefaction curves (threshold is 24,000) showing microbial diversity based on the Shannon index (upper panel) and Observed features (bottom panel) from Rag1−/− mice. [file 18_2022_4271_MOESM5_ESM.tif]

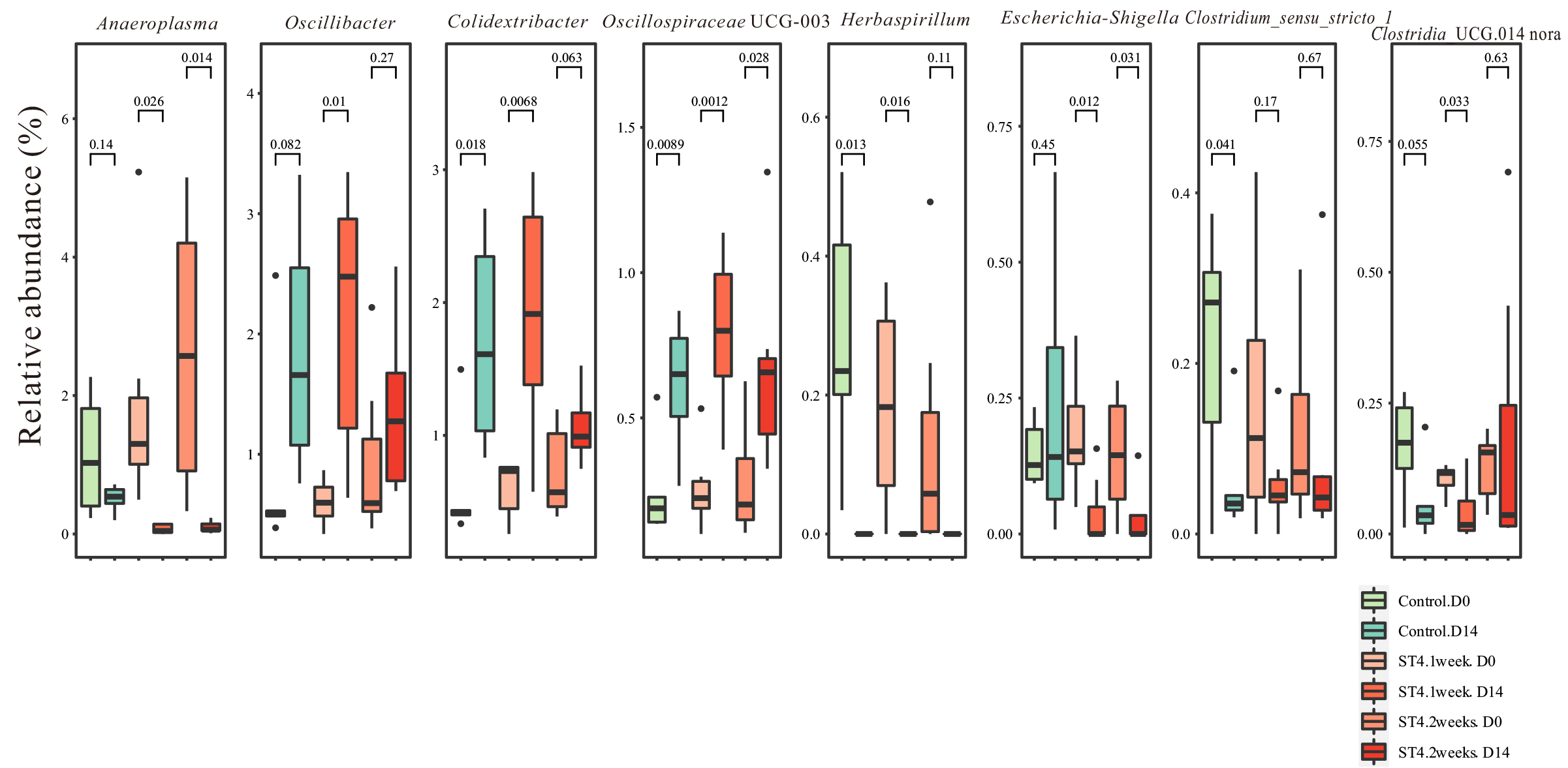

Supplement: Supplementary file 6 — Supplementary file6 (TIF 311 KB) Figure S6. Comparison of relative abundancies of different taxa between control and ST4-colonized mice. [file 18_2022_4271_MOESM6_ESM.tif]

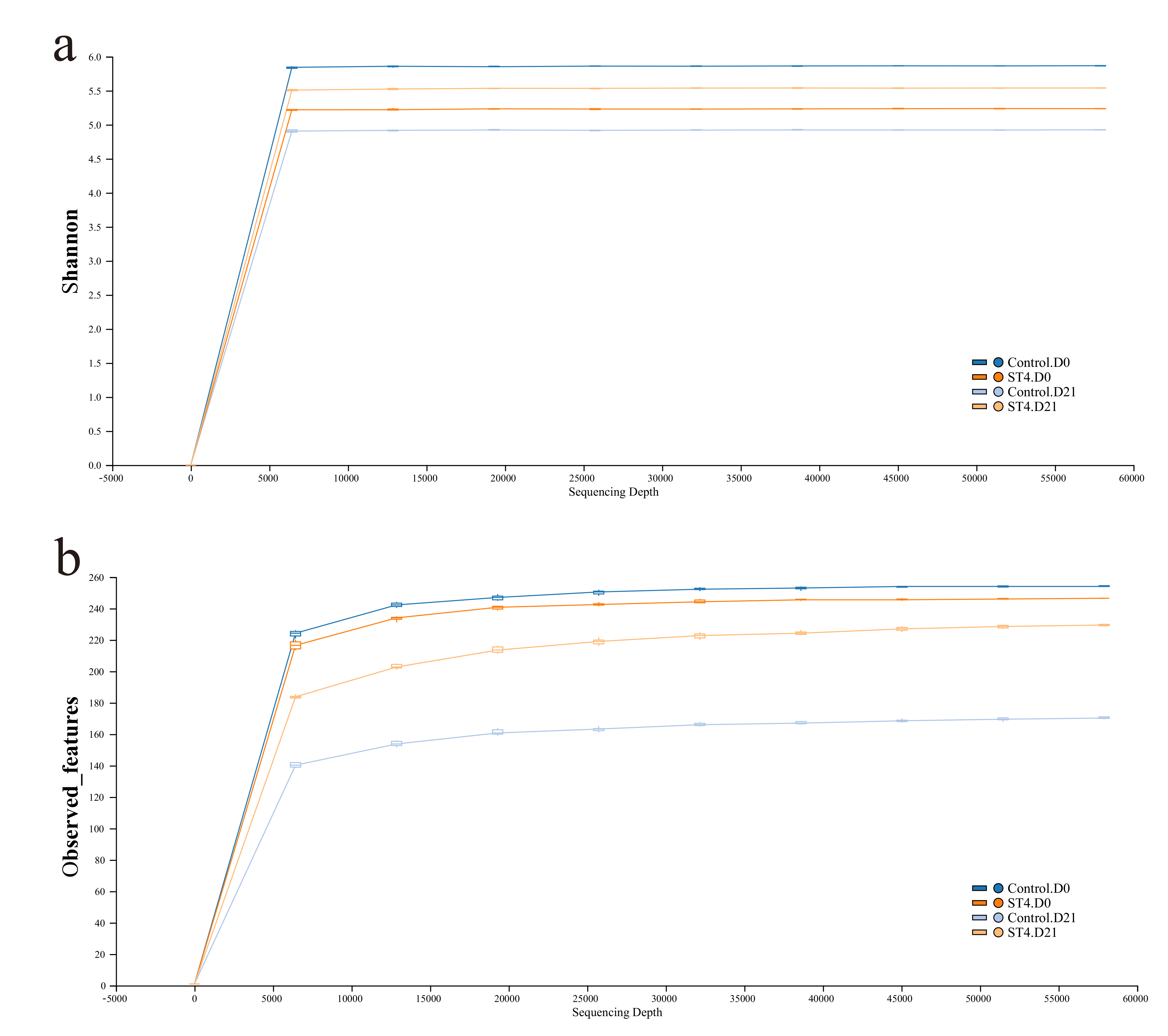

Supplement: Supplementary file 7 — Supplementary file7 (TIF 526 KB) Figure S7. Rarefaction curves (threshold is 58,000) showing microbial diversity based on the Shannon index (upper panel) and Observed features (bottom panel) from DSS-induced colitis mice. [file 18_2022_4271_MOESM7_ESM.tif]

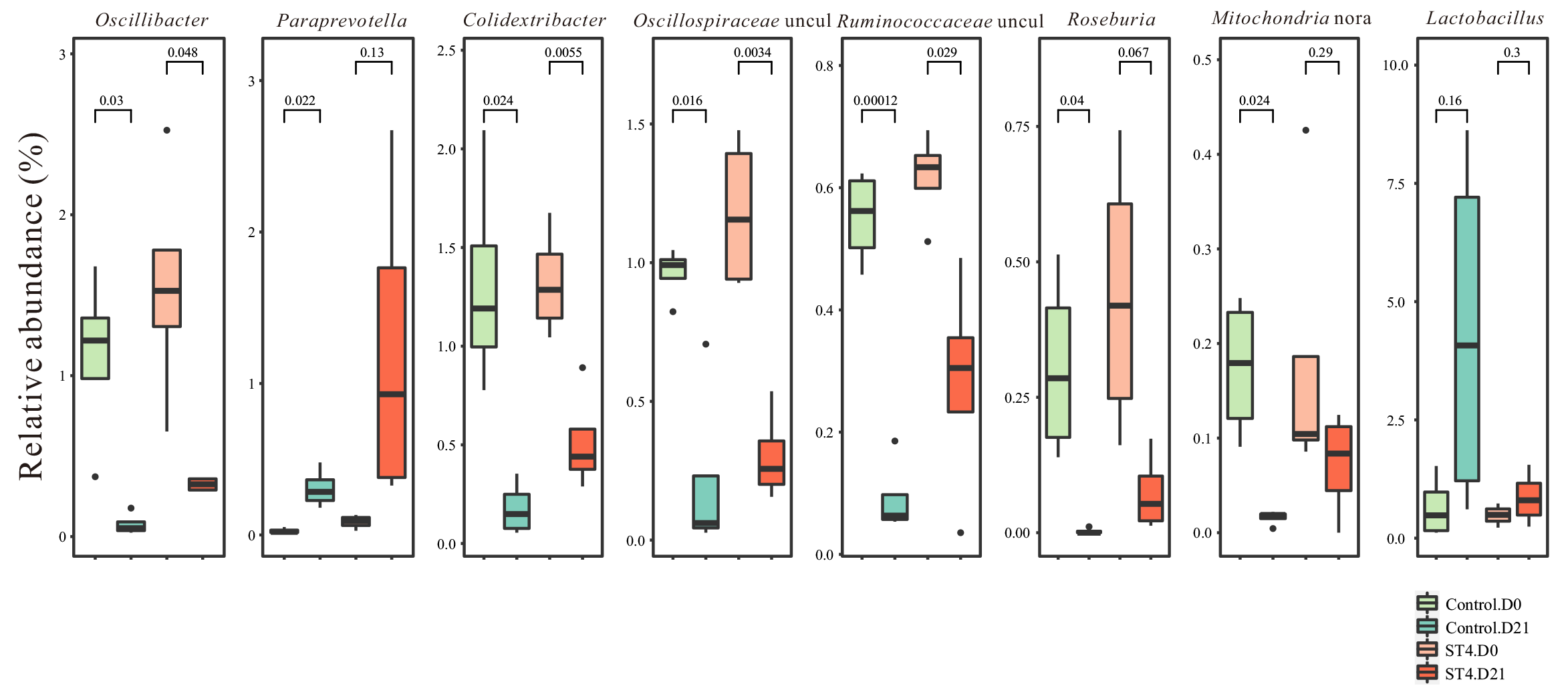

Supplement: Supplementary file 8 — Supplementary file8 (TIF 236 KB) Figure S8. Comparison of relative abundancies of different taxa between control and ST4-colonized mice. [file 18_2022_4271_MOESM8_ESM.tif]

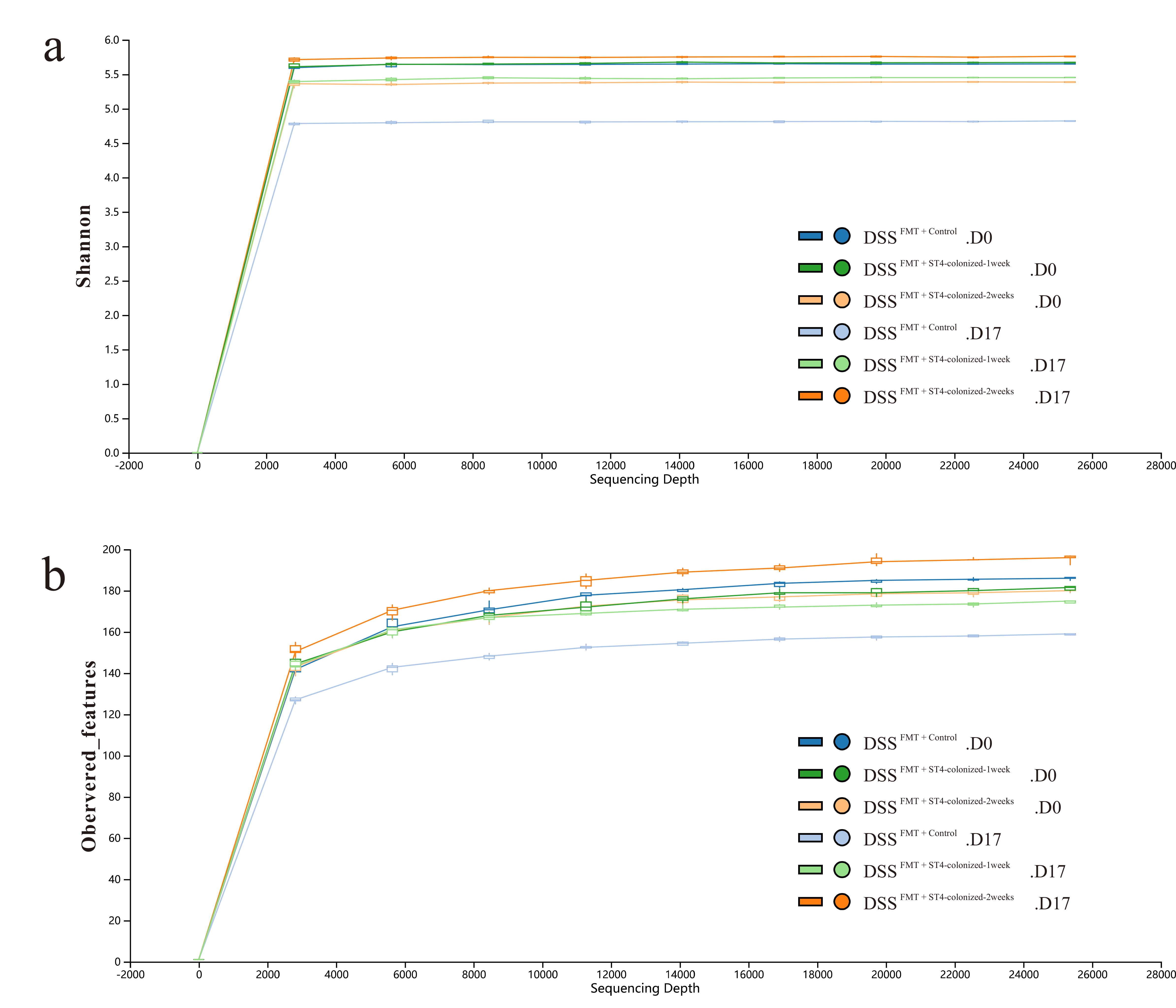

Supplement: Supplementary file 9 — Supplementary file9 (TIF 826 KB) Figure S9. Rarefaction curves (threshold is 26,000) showing microbial diversity based on the Shannon index (upper panel) and Observed features (bottom panel) from DSSFMT mice. [file 18_2022_4271_MOESM9_ESM.tif]

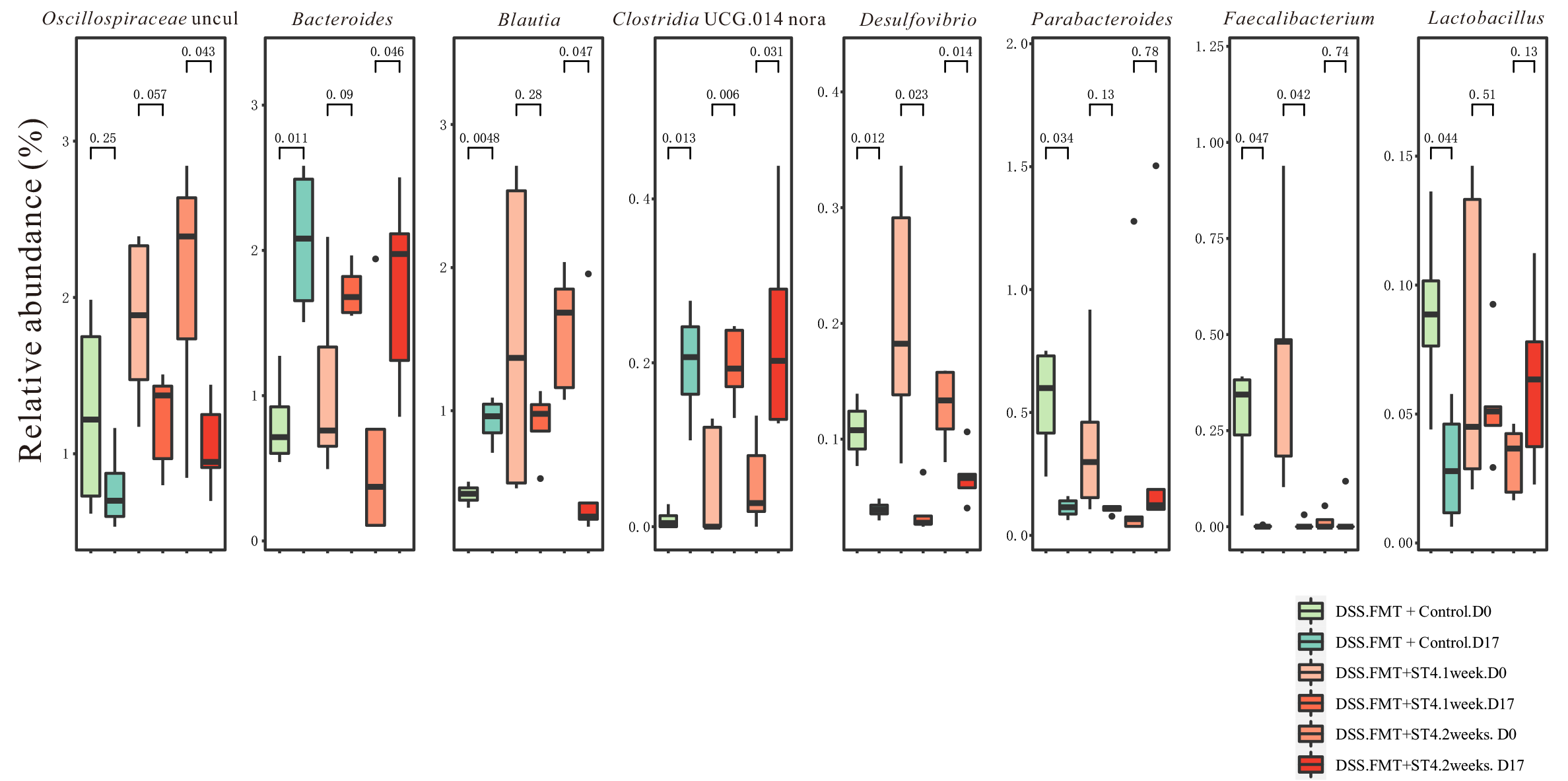

Supplement: Supplementary file 10 — Supplementary file10 (TIF 290 KB) Figure S10. Comparison of relative abundancies of different taxa between control and ST4-colonized mice. [file 18_2022_4271_MOESM10_ESM.tif]

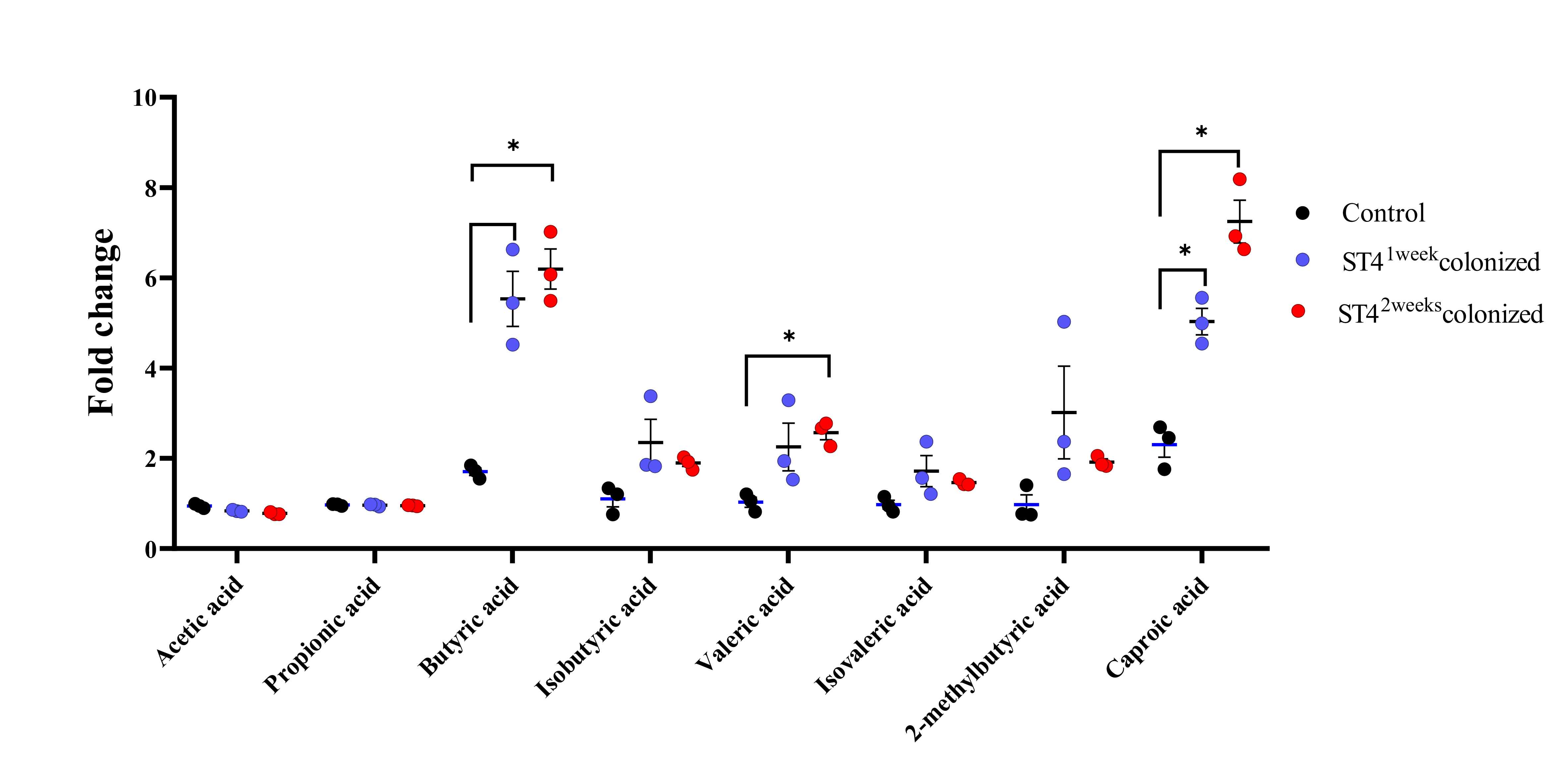

Supplement: Supplementary file 11 — Supplementary file11 (TIF 1457 KB) Figure S11. Fold-change of each SCFA relative to levels at day 0 from Rag1-/- mice (donor mice). [file 18_2022_4271_MOESM11_ESM.tif]
